# *TRIM17* promotes the progression of osteosarcoma by regulating *PDK1* m6A modification-mediated *AKT/mTOR* pathway activation through ubiquitination of *FTO*

**DOI:** 10.1038/s41419-025-08070-5

**Published:** 2025-10-27

**Authors:** Wenda Liu, Di Zheng, Xinghan Huang, Zhun Wei, Zicheng Wei, Weichun Guo

**Affiliations:** Department of Orthopaedics, Renmin Hospital of Wuhan University, Wuhan, Hubei Province China

**Keywords:** Bone cancer, Tumour biomarkers, Sarcoma

## Abstract

Osteosarcoma is an extremely aggressive malignant tumor, which is quite common among children and has a high rate of disability and mortality. Tripartite Motif Containing 17 (***TRIM17***) is a member of the ***TRIM*** protein family and exhibits E3 ubiquitin ligase activity. In recent years, ***TRIM17*** has been implicated in the development of various tumors, particularly in cancer cell clonability and survival potential and drug resistance; however, its regulatory mechanism in osteosarcoma progression remains poorly understood. We found that ***TRIM17*** was significantly upregulated in osteosarcoma tissues and cells. Survival analysis revealed that ***TRIM17*** was associated with poor prognosis in osteosarcoma patients. The higher the expression level of ***TRIM17***, the worse the prognosis. Its expression was an independent prognostic factor for osteosarcoma patients. The effects of ***TRIM17*** on osteosarcoma cell clonability and survival potential, migration, and invasion were assessed through phenotypic assays. The results showed that the downregulation of ***TRIM17*** significantly inhibited osteosarcoma cell clonability and survival potential, migration, and invasion, whereas its overexpression promoted these processes. ***FTO*** is an m6A methyltransferase and has been identified as a new target for ***TRIM17***. Mechanistically, ***TRIM17*** promotes the ubiquitination and degradation of ***FTO*** protein, enhances ***PDK1*** mRNA stability via N6-methyladenosine (m6A) modification, and subsequently promotes phosphorylation-dependent activation of the ***AKT/mTOR*** signaling pathway, thereby driving osteosarcoma malignancy. In summary, our findings suggest that ***TRIM17*** may serve as a potential prognostic marker and therapeutic target for osteosarcoma.

## Introduction

Osteosarcoma is the most common primary malignant bone tumor, predominantly occurring in the epiphysis of long bones. It is the second leading cause of cancer-related mortality among children and adolescents [1]. Osteosarcoma is highly metastatic, with early distant metastasis occurring easily, the most commonly metastatic organ is the lung [2]. Currently, limb salvage surgery combined with preoperative and postoperative adjuvant chemotherapy is the standard treatment for osteosarcoma [3]. However, for patients with distant metastasis, the current treatment options are ineffective, and the 5-year survival rate is less than 20% [4]. Tumor recurrence, distant metastasis, and chemotherapy resistance pose significant challenges in osteosarcoma treatment, contributing to the lack of substantial improvement in survival rates for patients with advanced osteosarcoma over the past decades [5]. It is of great significance to further study osteosarcoma at the genetic and molecular level and identify new therapeutic targets and molecular mechanisms.

The ***AKT/mTOR*** signaling pathway, as the core regulatory network for cell growth, proliferation, and metabolism, its abnormal activation is closely related to the malignant progression of various tumors [6, 7]. For example, in breast cancer, the activation of this pathway can promote cell migration [8]; in gastric cancer, its overactivation is associated with chemotherapy resistance [9]. In osteosarcoma, the abnormal activation of the ***AKT/mTOR*** pathway has been confirmed to be directly related to the invasive ability and lung metastasis potential of tumor cells [10]. However, the upstream regulatory mechanism of this pathway in osteosarcoma, especially the regulatory role of post-transcriptional modifications, still awaits in-depth study.

Tripartite motif 17 (***TRIM17***) is a member of the ***TRIM*** family of proteins, which are evolutionarily conserved and widely present in mammalian tissues and cells [11]. Structurally, the amino-terminal region of ***TRIM*** proteins contains a RING domain, one or two B-box domains, and a coiled-coil domain, and its carboxy-terminal domain is highly specific [12]. Functionally, most ***TRIM*** proteins possess E3 Ubiquitin ligase activity, regulate target protein degradation through the Ubiquitin proteasome system, and participate in biological processes such as cell clonability and survival potential, differentiation, and apoptosis [13]. It is also related to intracellular signal transduction, gene transcription, protein stabilization, and DNA damage repair. A large number of studies have shown that the expression and dysfunction of ***TRIM*** proteins play an important role in the occurrence and development of malignant tumors [14–16]. Limited literature has confirmed that ***TRIM17*** plays an important role in tumor progression. ***TRIM17*** is significantly upregulated in gastric cancer and is closely related to the prognosis of patients [17]. ***TRIM17*** plays a tumor-promoting role by promoting ubiquitination and degradation of ***BAX*** protein and inhibiting cell apoptosis. ***TRIM17*** is upregulated in cisplatin-resistant lung cancer tissues and cells and reduces cisplatin sensitivity of lung cancer cells by promoting ubiquitination and degradation of ***RBM38*** protein [18]. In addition, ***TRIM17*** can regulate the expression of ***MCL-1, ZSCAN21, BCL2A1****,* and other proteins, affecting apoptosis and autophagy [19–21]. However, the role of TRIM17 in the occurrence and development of osteosarcoma, as well as its specific mechanisms of action, remain to be further studied.

As an m6A demethylase, ***FTO*** regulates the stability and translation efficiency of mRNA by removing the N6-methyladenosine modification of RNA. In liver cancer and breast cancer, ***FTO*** promotes tumor progression by regulating the m6A level of oncogenes [22, 23], but its role in osteosarcoma and the upstream and downstream regulatory network remains unclear. Recent studies suggest that ***FTO*** may activate the ***AKT*** pathway by regulating the m6A modification of ***PDK1*** [24], but whether this mechanism is conconservative in osteosarcoma still needs to be verified.

In view of the carcinogenic effect of ***TRIM17*** in tumors, the key position of the ***AKT/mTOR*** pathway in osteosarcoma, and the potential function of ***FTO*** as a regulatory factor of m6A modification, we propose the hypothesis: ***TRIM17*** can activate the ***AKT/mTOR*** pathway and promote the progression of osteosarcoma by regulating ***FTO***-mediated ***PDK1*** m6A modification. In this study, we observed that ***TRIM17*** expression was significantly elevated in osteosarcoma tissues compared to adjacent normal tissues, as well as in osteosarcoma cells, and high ***TRIM17*** expression was associated with poor prognosis in osteosarcoma patients. Silencing the expression of ***TRIM17*** in osteosarcoma cells reduced their malignant characteristics, whereas overexpression of ***TRIM17*** had the opposite effect. Mechanistic studies revealed that ***TRIM17*** promotes ubiquitination and degradation of ***FTO***, enhances ***PDK1*** mRNA stability through m6A modification, activates the ***AKT/mTOR*** signaling pathway, and drives osteosarcoma cell clonability and survival potential and invasion, thereby contributing to osteosarcoma progression. In summary, this study elucidates the role of ***TRIM17*** in osteosarcoma progression and its underlying molecular mechanisms. ***TRIM17*** may represent a potential therapeutic target for the clinical management of osteosarcoma patients.

## Materials and methods

### Data acquisition

We utilized the Tumor Immune Estimation Resource database to compare ***TRIM17*** gene expression between tumor tissues and normal tissues. Using data from The Cancer Genome Atlas (TCGA) (https://portal.gdc.cancer.gov/projects/TCGA-COAD), we collected RNA sequencing data and clinical information from osteosarcoma patients, excluded samples lacking follow-up data, and ultimately included 86 patients for analysis.

### Clinical samples

In this study, we obtained 12 sets of normal and osteosarcoma tissue samples from Renmin Hospital of Wuhan University between 2020 and 2024. This sample size is based on the common settings used in similar studies for the initial verification of gene expression differences. Inclusion criteria: Patients diagnosed with osteosarcoma through pathological examination and with complete clinical data. Exclusion criteria: Patients with concurrent other malignant tumors, those who have received preoperative radiotherapy and chemotherapy, or those with severe underlying diseases. The collection and use of all human samples were approved by the Ethics Committee of Renmin Hospital of Wuhan University. All patients signed the informed consent form.

### Cell culture and transfection

The human osteosarcoma cell lines HOS, 143B, U2OS, and MG63 were purchased from Wuhan Procell Biotechnology Co., Ltd., and the human osteoblast cell line hFOB1.19 was obtained from the National Center for Cell Culture of China (Shanghai), all cell lines were found to be mycoplasma-negative. The cells were cultured in DMEM medium supplemented with 10% fetal bovine serum (FBS) and maintained in a humidified incubator at 37 °C with 5% CO_2_. ***TRIM17*** overexpression (***LV-TRIM17***), ***TRIM17*** knockdown (***shTRIM17***), and corresponding control lentiviral particles and overexpression plasmids were designed and purchased from OBiO (Shanghai, China), and transfection was performed following standard protocols. ***LV-TRIM17*** is based on the pLVX-IRES-ZsGreen1 vector and incorporates the full-length CDS sequence of ***TRIM17*** (NM_006347.4). The targeting sequence of ***shTRIM17*** is 5′-GCCTTATGCTGAAGATGAA-3′, which is cloned into the pLVshRNA2 vector. The negative control shNeg sequence is 5′-CCTAAGGTTAAGTCGCCCT-3′. Additionally, FTO siRNA was obtained from OBiO. The ***FTO*** overexpressed virus inserted the full-length CDS sequence of FTO (NM_138694.3). The sequence of ***si-FTO*** was 5′-GAAGACUGAAGCUGAAUAA-3′, and the sequence of the negative control siNC was 5′-UUCUCCGAACGUGUCACGU-3′. The DNA methylation inhibitor 3-Deazaadenosine (DAA, MedChemExpress, USA) treated cells at a concentration of 1 μM for 48 h to inhibit DNA methyltransferase [25, 26].

### Quantitative real-time PCR (qRT-PCR)

Each osteosarcoma cell line was cultured under standard conditions, and total RNA was extracted using TRIzol reagent (Invitrogen, Carlsbad, CA, USA). The RNA concentration and purity were quantified using the NanoDrop One/OneC ultramicro ultraviolet spectrophotometer (Thermo Fisher Scientific, USA). The extracted RNA was reverse-transcribed into cDNA using the Promega M-MLV kit (Promega, Beijing, China), and the resulting cDNA was used as a template for PCR amplification of ***TRIM17*** and the internal reference gene ***GAPDH***. The amplified products were analyzed using SYBR®Premix Ex Taq™ (Takara, Tokyo, Japan). The primers were as follows: ***TRIM17*** forward 5′-GACATGGAGTACCTTCGGGA-3′ and reverse 5′-GACATGGAGTACCTTCGGGA-3′; ***PDK1*** forward 5′-CTGTGATACGGATCAGAAACCG-3′ and reverse 5′-TCCACCAAACAATAAAGAGTGCT-3′; ***FTO*** forward 5′-ACTTGGCTCCCTTATCTGACC-3′ and reverse 5′- TGTGCAGTGTGAGAAAGGCTT-3′; ***GAPDH***, forward, 5′-CATGAGAAGTATGACAACAGCCT-3′, reverse, 5′-AGTCCTTCCACGATACCAAAGT-3′.

### Western blot

Cells in a healthy growth state were harvested, treated with RIPA lysis buffer (Servicebio, Wuhan, China), and lysed on ice for 30 min. The lysate was scraped and transferred to a centrifuge tube. The samples were centrifuged at 4 °C and 12,000 rpm for 15 min, and the supernatant was collected, mixed with 5× loading buffer, and boiled for 10 min to prepare the protein samples. Protein concentration was quantified using the BCA assay. Gels were prepared using a gel rapid configuration kit (Servicebio, Wuhan, China). Samples were placed in gel Wells for electrophoresis. The concentrated gel was subjected to electrophoresis at 70 V, and the separation gel was subjected to electrophoresis at 100 V to separate proteins. Remove the concentrated gel and cut the PVDF membrane. Pre-wet it with methanol and then soak it in the transfer solution for 2 to 5 min. Place the PVDF membrane and gel in the transfer interlayer and put it in the transfer tank. Add the pre-cooled transfer solution and electrically rotate it at 200 mA for 2 h under ice bath conditions. Put the PVDF membrane into the sealing solution (TBST containing 5% skimmed milk) and seal it at room temperature for 1 h. The PVDF membrane was rinsed with TBST and then placed in the primary antibody diluted with the blocking solution, and gently shaken at 4 °C overnight. Take the PVDF membrane out of the diluted primary antibody and wash the membrane with TBST for 3 × 15 min. Place the PVDF membrane into the secondary antibody diluted with TBST and incubate gently at room temperature for 2 h. Wash the PVDF membrane with TBST for 3 × 15 min. Finally, imaging and analysis were carried out using ECL luminescent liquid. The antibodies used in this study, along with their specific application details, are listed in Supplementary Table 1. Primary antibodies were diluted in 5% skim milk powder at the following concentrations: ***TRIM17*** (1:1000), ***GAPDH*** (1:10000), ***E-cadherin*** (1:1000), ***N-cadherin*** (1:500), ***Vimentin*** (1:20000), ***p-AKT T308*** (1:1000), ***t-AKT*** (1:1000), ***p-mTOR S2448*** (1:1000), ***t-mTOR*** (1:1000), ***p-S6K1 T389*** (1:2000), ***t-S6K1*** (1:1000), ***FTO*** (1:10000), ***Flag*** (1:2000), ***HA*** (1:1000), ***PDK1*** (1:10000).

### CCK-8 assay

The cells were cultured in 96-well plates with 1 × 10^5^ cells per well. Dilute the reagent in serum-free DMEM medium at a ratio of 1:10 (10 μL CCK-8 reagent (Servicebio, Wuhan, China, G1613-1ML) for every 100 μL of medium). Add 10 µL of CCK-8 diluent to each well. Return the 96-well plates to the incubator and incubate for 1–4 h. The same assays were performed at 24, 48, 72, 96, and 120 h. The optical density value of each well was measured at 450 nm using an enzyme labeler.

### colony formation assay

The cells were inoculated into 6-well plates at a density of 1000 cells per well and cultured in complete medium for 1–2 weeks until visible colonies were formed. Medium is updated every three days. After colony formation (diameter ≥ 0.5 mm), the cells were fixed at room temperature with 4% paraformaldehyde (PFA) for 15 min, and then washed three times with PBS. Then stain with 0.1% crystal violet solution for 30 min, and then rinse thoroughly with distilled water. The stained colonies were air-dried and images were captured with a digital camera. Colonies with a diameter of ≥ 0.5 mm were counted using ImageJ software to evaluate the clonability and survival potential of the cells.

### Scratch healing assay and transwell invasion assay

Osteosarcoma cells were seeded into a six-well plate. Upon reaching confluence, a 200 µL pipette tip was used to create a cross-shaped scratch in the center of each well. Serum-free medium was added to each well, and the cells were cultured for 36 h, after which they were observed and photographed. Images were processed and analyzed using Photoshop (PS) software. In the transwell invasion experiment, Matrigel (50–100 μL, BD Biosciences, USA) was pre-coated on the upper cavity membrane (pore size 8 μm, Corning, USA). The cells were trypsinized, resuspended in serum-free DMEM, and 2 × 10⁴ cells were inoculated into the upper chamber. 600 μL of DMEM containing 10% FBS was injected into the inferior cavity as a chemoattractant. After incubating the eggs at 37 °C for 24 h, the cells on the upper surface of the membrane were removed with a cotton swab. The cells that invaded the membrane were fixed with 4% PFA for 15 min, stained with 0.1% crystal violet for 30 min, and counted under a light microscope.

### RNA-Seq

The total RNA of osteosarcoma cells was extracted using TRIzol reagent. The purity (A260/A280 = 1.8–2.0) and integrity (RIN ≥ 8.0) were detected by NanoDrop and Agilent 2100 Bioanalyzer. The cDNA Library was constructed using the NEBNext Ultra RNA Library Prep Kit for Illumina, and double-ended sequencing (PE 150 bp) was performed on the Illumina HiSeq X Ten platform. After the sequencing data were quality controlled by FastQC, they were compared to the human genome (hg38) using HISAT2. The DESeq2 package was used for differential gene analysis (|log2FC| ≥1, *P* < 0.05), and the clusterProfiler package was used for KEGG enrichment analysis.

### MeRIP-Seq

Extract the total RNA of osteosarcoma cells and break the RNA to 100–200 nt using RNA Fragmentation Reagent (Thermo Fisher). Incubate with m6A antibody (Abcam, ab151230) and magnetic beads (Thermo Fisher, 88842). After adding fragmented RNA, shake at 4 °C overnight. After washing the magnetic beads, RNA is released using the elution buffer. cDNA libraries were constructed by immunoprecipitation of RNA and input RNA, respectively, and sequenced on the Illumina HiSeq platform (PE 150 bp). The m6A enrichment region was identified using the MeRIPseqR package. Motif analysis was performed using HOMER software. The screening criteria were Fold Change ≥ 2, *P* < 0.05.

### Docking technique

Download the three-dimensional structures of TRIM17 (ID: 6Z08) and FTO (ID: 5K9F) from the PDB database, and remove the ligands and water molecules. The amino acid residues were repaired with PyMOL and the Gasteiger charge was added. Use the FTO structure as a ligand and save it in the PDBQT format. The AutoDock Vina software was adopted to set the search space (central coordinates *x* = 10.2, *y* = 25.6, *z* = 32.4, grid size 20 × 20 × 20 A), and the energy threshold was set to −8.0 kcal/mol. Select the conformation with the lowest binding energy and visualize hydrogen bonds and hydrophobic interactions using PyMOL.

### Ubiquitination experiment in vivo

293 T cells were transfected with Flag-TRIM17 and HA-Ubiquitin plasmids. After 48 h, they were treated with MG132 (final concentration of 10 μM) for 4 h. Cells were collected and lysed in lysis buffer containing 1% NP-40 (50 mM Tris-HCl, pH 7.4, 150 mM NaCl, 1 mM EDTA), and ultrasonically fragmented (300 W, 3 × 10 s). Take the supernatant and incubate it with the Flag antibody at 4 °C overnight, then add the Protein A/G magnetic bead (Thermo Fisher) and incubate for 2 h. Wash the magnetic beads 5 times, add 5 × Loading Buffer and boil for 10 min. Western blot was used to detect HA-Ubiquitin and FTO.

### Protein co-inmunoprecipitation (Co-IP) assay

For the Co-IP assay, cells were resuspended in pre-cooled lysis buffer [50 mM Tris-HCl (pH 7.4), 150 mM NaCl, 1% NP-40, 0.5% sodium deoxycholate, 0.1% SDS, 1 mM EDTA, 1× protease inhibitor cocktail (Roche, USA), and 1% phenylmethylsulfonyl fluoride]. Cells were lysed on ice for 30 min, followed by centrifugation at 4 °C and 12,000 rpm for 15 min. The supernatant was collected, and protein concentration was quantified using the BCA assay. For immunoprecipitation, 800 μL of the protein supernatant was mixed with 5 μg of the corresponding primary antibody and incubated on a rotary shaker at 4 °C overnight. Pre-treated Protein A/G magnetic beads (Thermo Fisher Scientific, USA) were added and incubated at room temperature for 1 h. The supernatant was removed using a pipette, and the remaining immunoprecipitated (IP) sample, referred to as the “antigen-antibody-magnetic bead complex,” was collected. An appropriate volume of 5× loading buffer was added, and the sample was boiled for 10 min. After the protein co-immunoprecipitation (Co-IP) products were separated by SDS-PAGE, they were analyzed by liquid chromatography-mass spectrometry (LC-MS/MS). The mass spectrometry data were processed using MaxQuant software and compared with the UniProt human protein database to identify the specific binding proteins. For Co-IP, the following primary antibodies were used at 5 μg per 800 μL cell lysate: ***TRIM17***, ***FTO***, ***Flag***, ***HA***. Isotype-matched IgG was used as a negative control.

### Stability experiment

Cycloheximide (CHX) treatment: 24 h after cell inoculation, CHX (final concentration 100 μg/mL) was added. Cells were collected at 0, 2, 4, and 8 h respectively to detect the ***FTO*** protein level. ***GAPDH*** was used as the internal reference to calculate the protein half-life. The cells were treated with actinomycin D (5 μg/ml). After reaching a certain time point, the cells were collected and total RNA was extracted. Remove genomic DNA Reverse transcription was performed to synthesize cDNA and qPCR detection was carried out. Data analysis was conducted and the half-life of the target RNA was calculated.

### Dual-luciferase reporter assay

The recombinant plasmid was constructed by inserting the ***PDK1*** transcription initiation promoter region into the luciferase reporter gene plasmid (pGL3-basic). ***FTO***-specific siRNA (***si-FTO***) or the overexpression vector (pcDNA3.1-***FTO-HA***) was co-transfected with the recombinant plasmid into osteosarcoma cells. After 24 h, the cells were harvested, lysed using lysis buffer, and 20 μl of the cell lysate was transferred to a black luminometer plate. A 100 μL aliquot of 1× firefly luciferase reaction solution was added, gently shaken, and mixed thoroughly to measure firefly luciferase activity. Subsequently, 100 μL of 1× Renilla luciferase reaction solution was added to measure Renilla luciferase activity, and the ratio of firefly to Renilla luciferase activity was calculated as the final result.

### Immunohistochemistry (IHC)

Bake the paraffin slices in an oven at 60 °C for 30 min. Then, dewaxing with xylene I, xylene II, and xylene III for 5 min respectively, hydration with anhydrous ethanol, 95% ethanol, 85% ethanol, 70% ethanol and pure water for 5 min, and washing with phosphate-buffered saline (PBS). Then add the antigen repair solution, put the slices in, place it in the microwave oven on medium heat for 8 min, turn off the heat for 7 min, and then turn to low heat for 8 min. Take out the staining box and cool it to room temperature. Draw circles in immunohistochemistry to prevent the reagent from flowing out. Add endogenous peroxidase blocking solution dropby, incubate at room temperature for 10 min, and wash three times with PBS buffer, each time for 5 min. Drip the blocking serum, incubate at 37 °C for 30 min and discard the excess serum. Drip the primary antibody, incubate in a wet box at 37 °C for 2 h, and wash three times with PBS buffer, each time for 5 min. Add horseradish peroxidase coupled with secondary antibody (diluted at 1:200, Zhongshan Jinqiao, China), incubate at 37 °C for 30 min, wash three times with PBS buffer, each time for 5 min. For color and imaging development, the sections were incubated with 3,3′-diaminobenzidine solution (Zhongshan Jinqiao, China) at room temperature for 5–10 min. Under microscopic observation, the chromogenic development was stopped when the positive results were significantly enhanced and the background was clean. Add hematoxylin staining solution and incubate at room temperature for 5 min. Rinse with tap water and it turns blue again. 70%–85%–95%–100% alcohol gradient dehydration, 5 min each time, three transparent tanks of xylene, 2 min each tank, neutral gum sealing sheet. Primary antibodies for IHC were diluted in 5% BSA as follows: ***TRIM17*** (1:200), ***E-cadherin*** (1:200), ***N-cadherin*** (1:100), ***p-AKT T308*** (1:200), ***t-AKT*** (1:200), ***p-mTOR S2448*** (1:200), ***t-mTOR*** (1:200), ***p-S6K1 T389*** (1:400), ***FTO*** (1:2000), ***Flag*** (1:400), ***HA*** (1:200), ***PDK1*** (1:2000).

### Animal models

For the subcutaneous (SC) tumor model, 4-week-old male nude mice (Shulaibao Biotechnology Co., Ltd., Wuhan, China. The mice were randomly divided into different groups using a random number table. Six nude mice were assigned to each group to meet the sample size requirements for independent sample *t*-tests and one-way analysis of variance) were subcutaneously injected with 1 × 10⁶ wild-type 143B cells or ***shTRIM17***-transfected 143B cells (resuspended in 100 μL PBS) into the right flank. Tumor growth at the inoculation site was monitored regularly, and the long and short diameters of the SC tumors were measured to evaluate tumor size and morphological changes. Four weeks post-inoculation, the mice were anesthetized, dissected, and subjected to subsequent analysis. Tumor volume (mm^3^) = (long diameter × short diameter^2^/2). For the lung metastasis model, we transfected lentivirus carrying firefly luciferase (pLVX-ShRNA2-Puro-Luciferase) into osteosarcoma cells, screened them with purinomycin, and collected well-growing tumors that were resuspended in PBS. The collected cells were injected into mice through the tail vein, with 200 μL for each mouse, that is, 1 × 10^6^ cells. The signal intensity was regularly detected by a live imaging system after vaccination. Two weeks later, the substrate luciferase was intraperitoneally injected at a dose of 150 mg/kg body weight. Fifteen minutes later, 0.3% pentobarbiturate sodium solution was intraperitoneally injected, and in vivo imaging was performed using the darkroom imaging platform IVIS Lumina III (PerkinElmer, USA) to observe the distribution and extent of lung metastases. The experimental results were recorded and analyzed by independent laboratory personnel who were not involved in the grouping and vaccination procedures. All animal assayal studies were approved by the Medical Ethics Committee of Renmin Hospital of Wuhan University.

### Statistical analysis

Statistical analyses were performed using GraphPad Prism 8.0 and R (version 4.0.1). Measurement data are expressed as mean ± standard deviation (SD). Differences between two groups were assessed using Student’s *t*-test or the Wilcoxon test, whereas differences among multiple groups were analyzed using one-way ANOVA. All assays were performed in triplicate, and a *P*-value < 0.05 was considered statistically significant.

## Results

### TRIM17 is highly expressed in osteosarcoma and is associated with poor prognosis

**Fig. 1:**
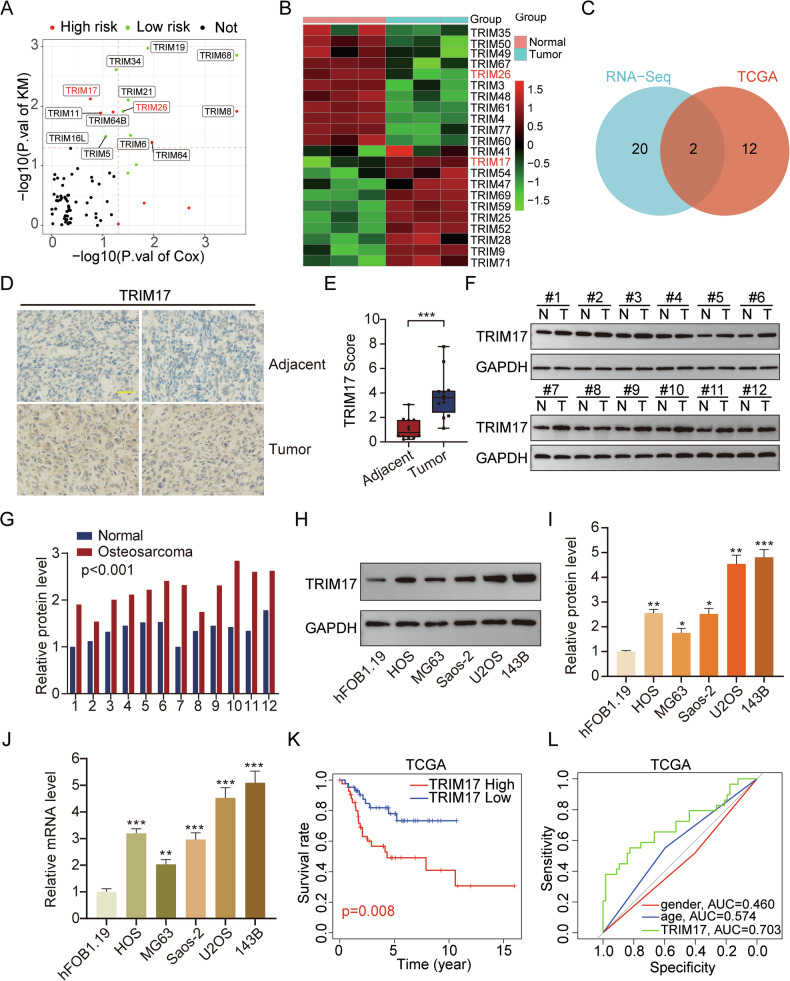
*TRIM17* is highly expressed in osteosarcoma tissues and cells and is negatively correlated with patient prognosis. **A** Volcanic maps show ***TRIM*** family genes associated with prognosis in patients with osteosarcoma. **B** RNA-Seq analysis of ***TRIM*** genes differentially expressed in osteosarcoma tissues and adjacent normal tissues. **C** Venn diagram of differentially expressed ***TRIM*** family genes. **D, E** ***TRIM17*** expression levels in osteosarcoma tissues and adjacent normal tissues were detected by IHC. **F**, **G** ***TRIM17*** expression levels in osteosarcoma tissues and adjacent normal tissues were detected by Western blot. **H–J** ***TRIM17*** expression levels in normal osteoblasts and osteosarcoma cell lines. **K** Kaplan–Meier survival analysis of ***TRIM17*** gene. **L** ROC curve was used to analyze the prognosis accuracy of ***TRIM17***. Student’s *t*-test (two groups) and one-way analysis of variance (more than two groups) were used to analyze the differences between groups. All data are expressed as mean ± standard deviation (SD). * *P* < 0.05, ** *P* < 0.01, *** *P* < 0.001.

By integrating transcriptome data from osteosarcoma samples in TCGA database and patient clinical information, Kaplan–Meier survival analysis was conducted, revealing that a subset of ***TRIM*** genes was associated with osteosarcoma patient prognosis (Fig. 1A). Additionally, transcriptome sequencing of osteosarcoma and adjacent normal tissue samples collected from our hospital revealed differential expression of certain ***TRIM*** genes in osteosarcoma (Fig. 1B). Venn diagram analysis of the survival and differential expression results demonstrated that ***TRIM26*** and ***TRIM17*** were differentially expressed in osteosarcoma and correlated with patient prognosis (Fig. 1C). As the mechanism of ***TRIM26*** in osteosarcoma has been previously investigated, this study focuses on exploring the role of ***TRIM17*** in osteosarcoma [14]. We used an online platform to analyze ***TRIM17*** expression differences between multiple tumors and normal tissues (Supplementary Fig. S1A). The results showed that ***TRIM17*** was upregulated in bladder urothelial carcinoma, cholangiocarcinoma, esophageal carcinoma, liver hepatocellular carcinoma, lung adenocarcinoma, lung squamous cell carcinoma, rectum adenocarcinoma, stomach adenocarcinoma, thyroid carcinoma, and uterine corpus endometrial carcinoma, but downregulated in kidney chromophobe, kidney renal clear cell carcinoma, Kidney renal papillary cell carcinoma, and Prostate adenocarcinoma. To further verify the role of ***TRIM17*** in osteosarcoma, we conducted immunohistochemical analysis on 12 groups of osteosarcoma tissues and adjacent normal tissues collected in the hospital (the results of two representative groups of immunohistochemistry are shown in the figure). The results indicated that the expression of ***TRIM17*** in osteosarcoma tissues was increased compared with adjacent normal tissues (Fig. 1D, E). Meanwhile, Western blot results showed that ***TRIM17*** protein levels in osteosarcoma tissues were significantly higher than those in adjacent normal tissues (Fig. 1F, G). In addition, the ***TRIM17*** protein and mRNA levels in osteosarcoma cell lines (HOS, MG63, Saos-2, U2OS, 143B) were significantly higher than those in normal osteoblasts (hFOB1.19) (Fig. 1H–J). Kaplan–Meier survival analysis showed that high expression of ***TRIM17*** was associated with shorter overall survival (Fig. 1K). ROC curve analysis showed that ***TRIM17*** expression level could accurately predict the prognosis of osteosarcoma patients (AUC: 0.703) (Fig. 1L). These findings suggest that ***TRIM17*** is highly expressed in osteosarcoma, and its elevated expression is associated with poor patient prognosis, indicating its potential as an independent prognostic marker.

### TRIM17 knockdown inhibited clonability and survival potential, migration, and invasion of osteosarcoma cells

**Fig. 2:**
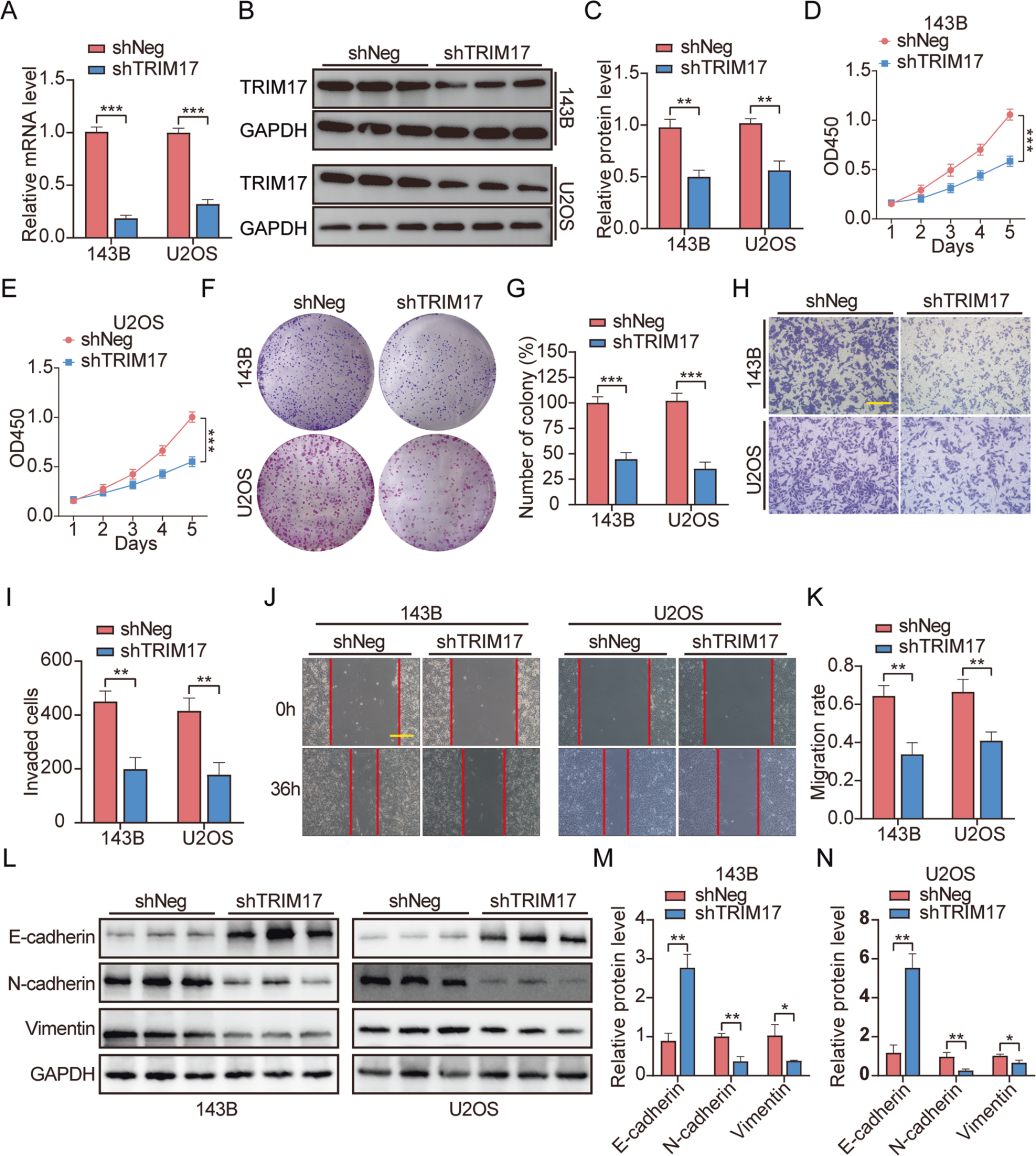
*TRIM17* knockdown inhibited clonability and survival potential, migration, and invasion of osteosarcoma cells. **A–C** The expression level of ***TRIM17*** in knockdown stable cells was verified. **D–G** The effect of ***TRIM17*** knockdown on cell clonability and survival potential was detected by CCK-8 and colony formation assay, scale bar: 200 μm. **H**, **I** Transwell invasion assay was used to detect the effect of ***TRIM17*** knockdown on cell invasion ability, scale bar: 200 μm. **J**, **K** The effect of ***TRIM17*** knockdown on cell migration was detected by wound healing assay, scale bar: 200 μm. **L–N** Effect of ***TRIM17*** knockdown on expression of EMT-related proteins. Student’s *t*-test (two groups) and one-way analysis of variance (more than two groups) were used to analyze the differences between groups. All data are expressed as mean ± standard deviation (SD). * *P* < 0.05, ** *P* < 0.01, *** *P* < 0.001.

Following lentiviral infection, a stable ***TRIM17*** knockdown cell line was established. qRT-PCR and Western blot analyses demonstrated that ***TRIM17*** mRNA and protein levels in ***shTRIM17*** cells were significantly reduced compared to the control group (***shNeg***) (Fig. 2A–C). CCK-8 and plate cloning assays showed that knockdown of ***TRIM17*** significantly inhibited cell clonability and survival potential (Fig. 2D–G). The transwell invasion assay showed that knockdown of ***TRIM17*** significantly reduced cell invasion (Fig. 2H, I). Wound healing assays showed that knocking down ***TRIM17*** significantly inhibited cell migration (Fig. 2J, K). The expression of epithelial-mesenchymal transition (EMT)-associated proteins was evaluated. Western blot analysis revealed a significant increase in ***E-cadherin*** levels and a significant decrease in ***N-cadherin*** and ***Vimentin*** levels (Fig. 2L–N). In conclusion, knockdown of ***TRIM17*** can significantly inhibit the clonability and survival potential, migration, and invasion of osteosarcoma in vitro.

### Overexpression of TRIM17 promotes clonability and survival potential, migration, and invasion of osteosarcoma cells

**Fig. 3:**
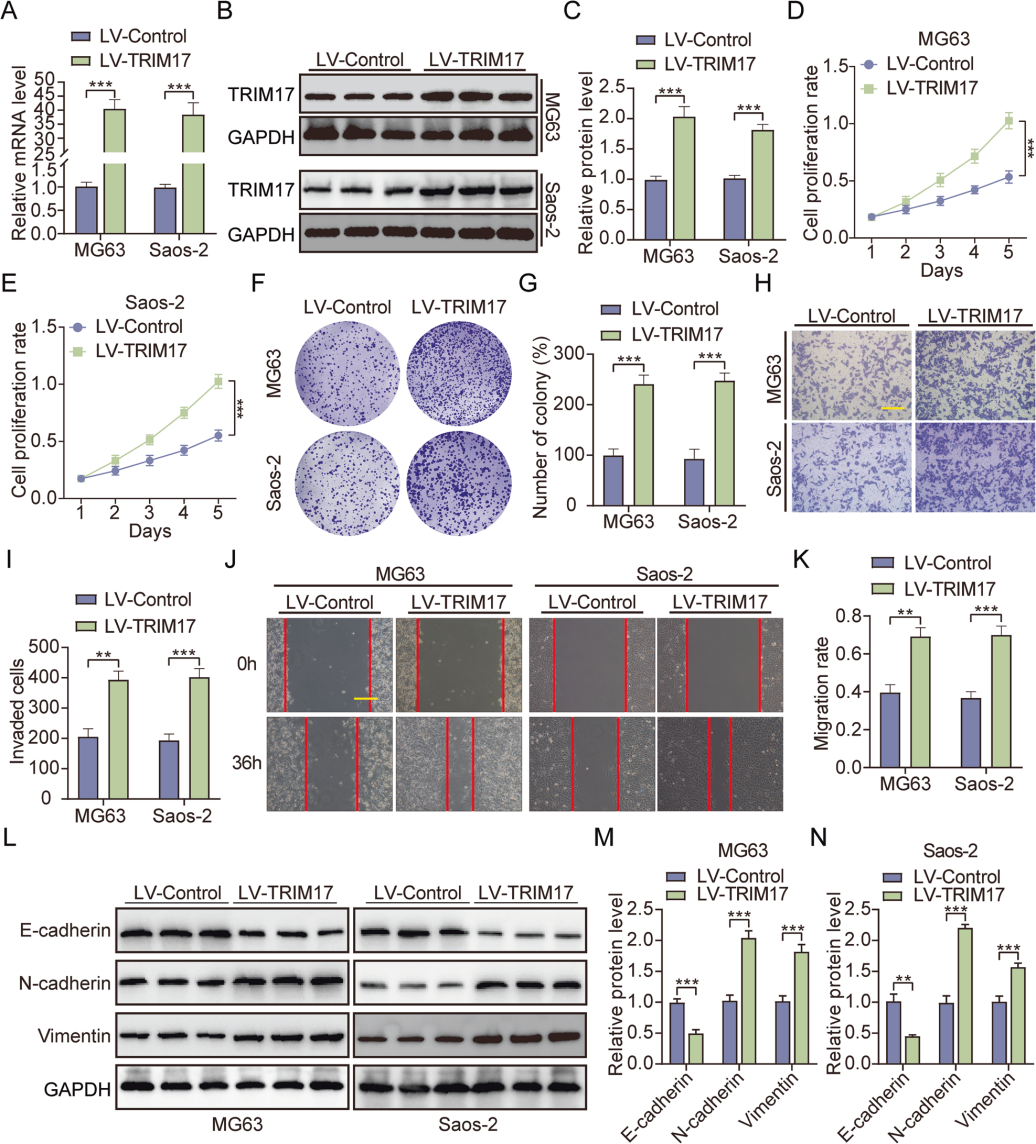
Overexpression of *TRIM17* promotes clonability and survival potential, migration, and invasion of osteosarcoma cells. **A–C** The expression level of ***TRIM17*** in overexpressed stable cells was verified. **D–G** The effect of overexpression of ***TRIM17*** on cell clonability and survival potential was detected by CCK-8 and colony formation assay, scale bar: 200 μm. **H**, **I** Transwell invasion assay was used to detect the effect of overexpression of ***TRIM17*** on cell invasion ability, scale bar: 200 μm. **J**, **K** The effect of overexpression of ***TRIM17*** on cell migration was detected by wound healing assay, scale bar: 200 μm. **L–N** Effect of overexpression of ***TRIM17*** on expression of EMT-related proteins. Student’s *t*-test (two groups) and one-way analysis of variance (more than two groups) were used to analyze the differences between groups. All data are expressed as mean ± standard deviation (SD). ** *P* < 0.01, *** *P* < 0.001.

A stable ***TRIM17***-overexpressing cell line was established using lentiviral transfection. qRT-PCR and Western blot analyses confirmed that ***TRIM17*** mRNA and protein levels in the overexpression group (***LV-TRIM17***) were significantly elevated compared to the control group (***LV-Control***) in the 143B, U2OS, MG63, and Saos-2 cell lines (Fig. 3A–C and Supplementary Fig. S2A–C). The results of CCK-8 and colony formation assays showed that overexpression of ***TRIM17*** could significantly promote cell clonability and survival potential (Fig. 3D–G and Supplementary Fig. S2D–G). The results of transwell invasion assay showed that overexpression of ***TRIM17*** could significantly promote the invasion ability of cells (Fig. 3H, I and Supplementary Fig. S2H, I). The results of wound healing assay showed that overexpression of ***TRIM17*** could significantly promote cell migration (Fig. 3J, K and Supplementary Fig. S2J, K). We performed Western blot assays to evaluate the expression of proteins associated with EMT. Western blot results showed that ***E-cadherin*** levels significantly decreased, while ***N-cadherin*** and ***Vimentin*** levels significantly increased (Fig. 3L–N and Supplementary Fig. S2L–N). In conclusion, regardless of whether the basal expression level of ***TRIM17*** in osteosarcoma cells is high or low, overexpression of ***TRIM17*** can significantly promote the clonability and survival potential, migration, and invasion of osteosarcoma in vitro.

### TRIM17 gene influences the progression of osteosarcoma by regulating the activity of AKT/mTOR signaling pathway

**Fig. 4:**
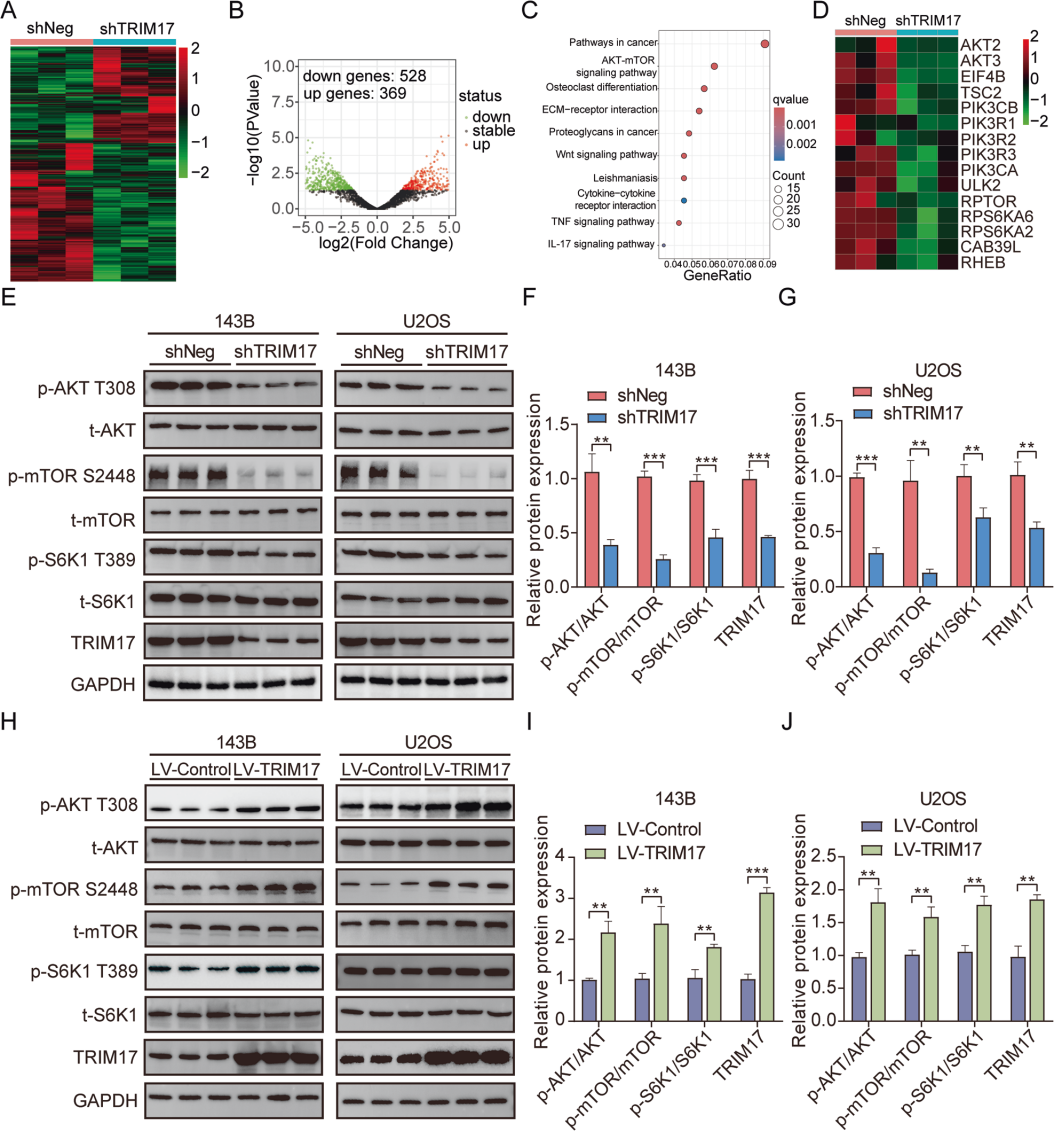
*TRIM17* knockdown inhibits *AKT/mTOR* signaling pathway activity. **A**, **B** Differentially expressed genes after silencing ***TRIM17*** gene expression. **C** KEGG enrichment analysis. **D** Effect of silencing ***TRIM17*** on ***AKT/mTOR*** pathway gene expression. **E–G** Effect of silencing ***TRIM17*** on ***AKT/mTOR*** pathway protein phosphorylation. **H–J** Effects of overexpression of ***TRIM17*** on ***AKT/mTOR*** pathway protein phosphorylation. Student’s *t*-test (two groups) and one-way analysis of variance (more than two groups) were used to analyze the differences between groups. All data are expressed as mean ± standard deviation (SD). ** *P* < 0.01, *** *P* < 0.001.

To investigate the specific mechanism of ***TRIM17*** in osteosarcoma progression, RNA sequencing (RNA-Seq) was performed on 143B cells from the ***TRIM17*** knockdown group and the control group. Heatmaps were generated to visualize the expression patterns of differentially expressed genes across the cell samples (Fig. 4A). The expression levels of a total of 897 genes were altered, among which 369 genes were upregulated in the ***TRIM17*** knockdown group, while the expression of another 528 genes was downregulated (Fig. 4B). Then, we performed functional enrichment analysis of differentially expressed genes (KEGG), and the results showed that more differentially expressed genes were enriched in the ***AKT-mTOR*** signaling pathway (Fig. 4C). At the same time, genes associated with the ***AKT-mTOR*** signaling pathway were downregulated after silencing ***TRIM17*** (Fig. 4D). Studies have shown that the ***AKT-mTOR*** signaling pathway plays an important role in tumor progression, transformation and metabolism, and is related to the malignancy of osteosarcoma [27, 28]. Based on these findings, we hypothesized that ***TRIM17*** modulates osteosarcoma progression by regulating the ***AKT-mTOR*** signaling pathway. To test this hypothesis, we examined changes in the expression of ***AKT-mTOR*** signaling pathway components following ***TRIM17*** knockdown and overexpression. Western blot analysis revealed that ***TRIM17*** knockdown significantly reduced the phosphorylation levels of ***AKT/mTOR*** pathway proteins (***AKT, mTOR, and S6K1***), whereas ***TRIM17*** overexpression increased their phosphorylation levels (Fig. 4E–J). These results suggest that ***TRIM17*** regulates the activation of ***AKT-mTOR*** signaling pathway in osteosarcoma.

### TRIM17 interacts with FTO and co-regulates the AKT/mTOR signaling pathway

**Fig. 5:**
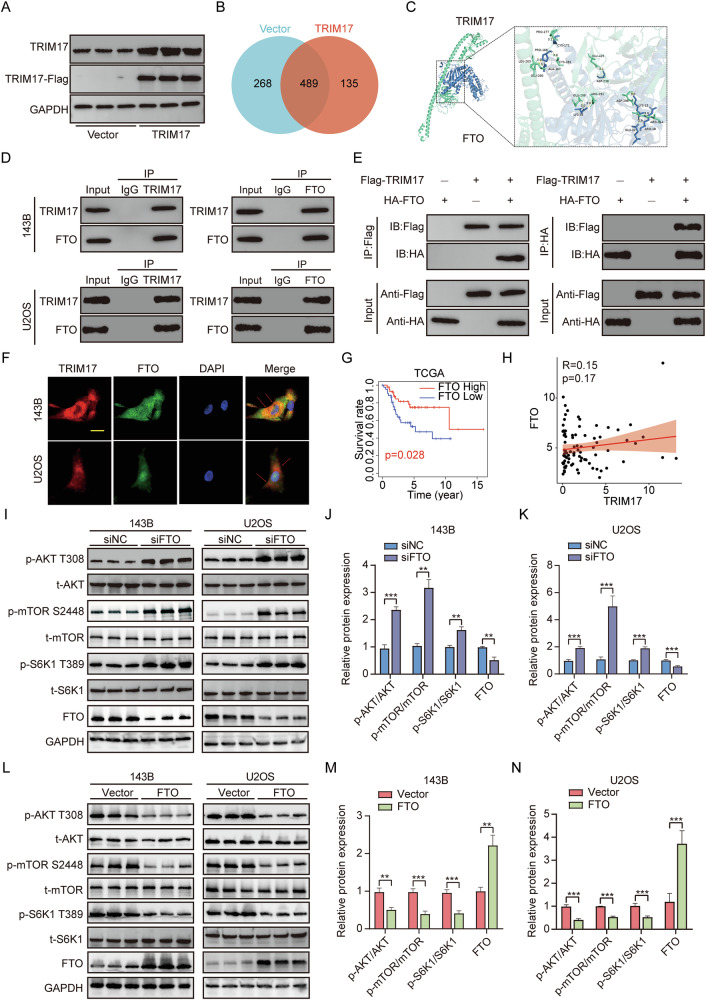
*TRIM17* interacts with *FTO* and *FTO* regulates the *AKT/mTOR* signaling pathway. **A** Overexpression efficiency of 293T cells transfected with plasmid. **B** The interacting proteins of ***TRIM17*** were identified by IP-MS. **C** Molecular docking predicts the interaction between ***TRIM17*** and ***FTO***. **D** Endogenous Co-IP of ***TRIM17*** and ***FTO*** in untransfected 143B and U2OS cells. IgG was used as a negative control. **E** The interaction between ***TRIM17*** and ***FTO*** in 293T cells co-transfected with ***TRIM17*** and ***FTO*** overexpressing plasmids was detected by Co-IP assay. **F** The co-localization of ***TRIM17*** and ***FTO*** was detected by cellular immunofluorescence, scale bar: 10 μm. **G** Kaplan–Meier survival analysis of ***FTO*** gene. **H** Correlation between ***TRIM17*** and ***FTO*** in TCGA osteosarcoma dataset. **I–K** Effect of silencing ***FTO*** on ***AKT/mTOR*** pathway protein phosphorylation. **L–N** Effects of overexpression of ***FTO*** on ***AKT/mTOR*** pathway protein phosphorylation. Student’s *t*-test (two groups) and one-way analysis of variance (more than two groups) were used to analyze the differences between groups. All data are expressed as mean ± standard deviation (SD). ** *P* < 0.01, *** *P* < 0.001.

After transfecting the Vector and ***TRIM17*** overexpression plasmid into 293T cells, the overexpression efficiency of ***TRIM17*** in 293T cells was verified by Western blot (Fig. 5A). A total of 135 specific binding proteins were identified in the ***TRIM17*** overexpression group by mass spectrometry after protein immunoprecipitation in the two types of cells (Fig. 5B). Among them, demethylase ***FTO*** may bind ***TRIM17*** specifically (Table 1). Using a molecular docking technique, it is predicted that ***TRIM17*** may bind to the ***FTO*** protein (Fig. 5C). To verify whether ***TRIM17*** and ***FTO*** can interact, we transfected ***TRIM17*** and ***FTO*** plasmids into 143B, U2OS, and 293T cells, respectively. Endogenous co-immunoprecipitation (Co-IP) in untransfected 143B and U2OS cells demonstrated the interaction between native ***TRIM17*** and ***FTO*** (Fig. 5D). Notably, this experiment was performed without exogenous ***TRIM17*** transfection, allowing detection of basal ***FTO*** levels, which are partially degraded by endogenous ***TRIM17*** but remain sufficient for Co-IP validation. Similarly, we transfected flag-labeled ***TRIM17*** and HA-labeled ***FTO*** into 293T cells, and the results also showed that ***TRIM17*** interacts with ***FTO*** (Fig. 5E). The results of cell immunofluorescence showed that ***TRIM17*** and ***FTO*** were co-localized in osteosarcoma cells (Fig. 5F). Moreover, Kaplan–Meier survival analysis showed that low ***FTO*** expression was associated with shorter overall survival in osteosarcoma patients (Fig. 5G). But the correlation analysis of osteosarcoma patients in the TCGA dataset showed that there was no correlation between ***TRIM17*** and ***FTO*** gene expression levels (Fig. 5H). We demonstrated that ***TRIM17*** interacts with ***FTO***. To further explore the role of ***FTO*** in ***TRIM17***-mediated osteosarcoma progression, ***FTO*** was knocked down or overexpressed in 143B and U2OS cells, respectively, and changes in the ***AKT-mTOR*** signaling pathway were assessed. Western blot analysis revealed that silencing ***FTO*** enhanced the phosphorylation of ***AKT-mTOR*** pathway proteins and activated the ***AKT-mTOR*** signaling pathway (Fig. 5I–K). Conversely, upregulation of ***FTO*** expression can inhibit the activation of the ***AKT-mTOR*** signaling pathway (Fig. 5L–N). In summary, ***TRIM17*** can interact with ***FTO***, and ***FTO*** may regulate the function of ***TRIM17*** in osteosarcoma by influencing the activation of the ***AKT-mTOR*** signaling pathway.

**Table 1:** The top ten proteins in the protein profile identification results.

Protein	Protein Q-score	Peptide Seqs
TRIM17	224.6804524	CRESREHRLHRV; YESRQRRYLGS; LDFEAGEVS
KIF11	168.5262356	ASAHSIVECDPVR; AVDQHNAEAQDIFGK
FTO	166.1621636	NEILTAILASLTAR: GSNIKHTEAEI
PRS6B	115.9913521	AVAHHTTAAFIR; EAVELPLTHFELYK
PRS4	106.4645135	GVILYGPPGTGK; IETLDPALIRPGR
PLST	102.2312906	QFVTPADVVSGNPK; YTLNVLEDLGDGQK
SAHH	101.9944747	VADIGLAAWGR: YPQLLPGIR
SMC4	99.06413846	LPQTEQELK; SLVHDLFQK; SNNIINETTTR
TRIP6	93.99349491	GQHFYAVER; GTPGPPPAHGAALQPHPR
PSD13	90.04594809	ITVNEVELLVMK; LNIGDLQVTK

### TRIM17 regulates the AKT/mTOR signaling pathway through ubiquitination of FTO protein, which affects the progression of osteosarcoma

**Fig. 6:**
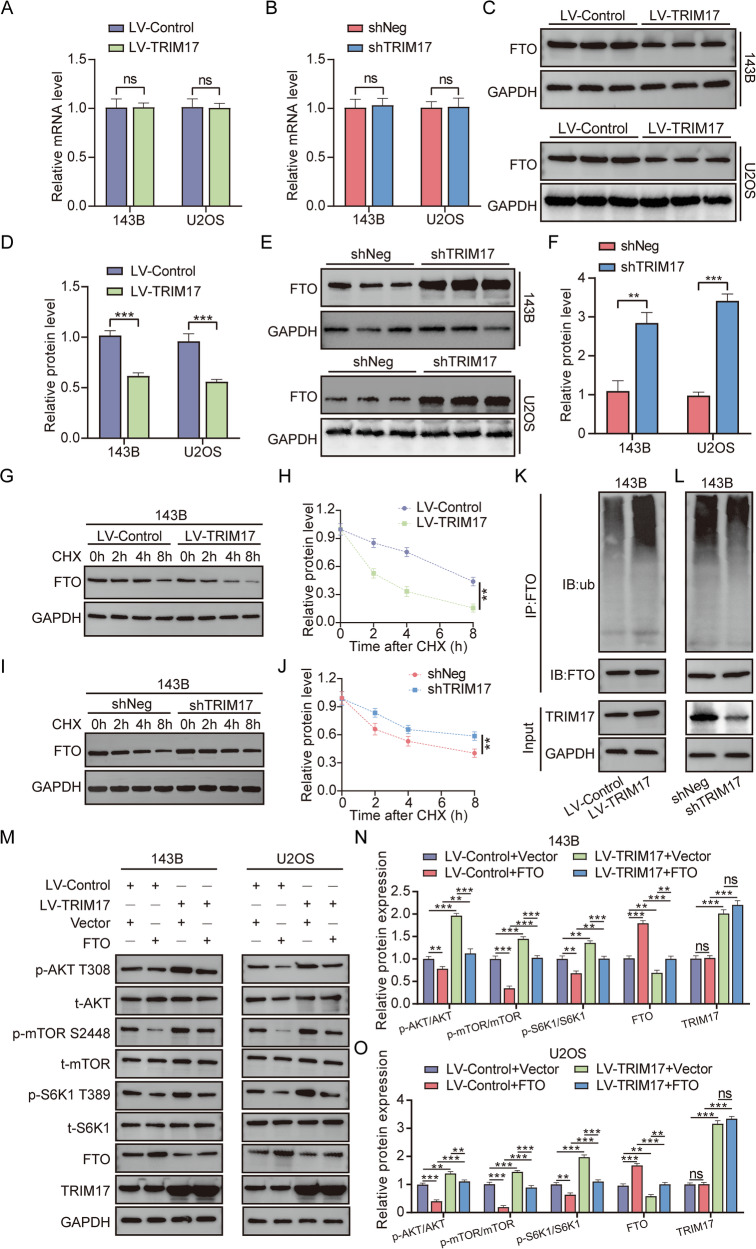
*TRIM17* promotes ubiquitination and degradation of *FTO* protein. **A**, **B** The effect of overexpression of ***TRIM17*** on ***FTO*** mRNA was detected by qRT-PCR. **C**, **D** Effect of overexpression of ***TRIM17*** on the level of ***FTO*** protein. **E**, **F** Effect of silencing ***TRIM17*** on ***FTO*** protein levels. **G–J** Cells were treated with CHX (100 μg/mL) and collected at 0, 2, 4, and 8 h. The ***FTO*** protein level was detected and the kinetics of protein degradation was analyzed. **K**, **L** Effect of ***TRIM17*** on ubiquitination level of ***FTO*** protein. **M–O** Overexpression of ***FTO*** regulates the effects of ***TRIM17*** on ***AKT/mTOR*** signaling pathway. Student’s *t*-test (two groups) and one-way analysis of variance (more than two groups) were used to analyze the differences between groups. All data are expressed as mean ± standard deviation (SD). ** *P* < 0.01, *** *P* < 0.001.

Previous studies confirmed that ***TRIM17*** and ***FTO*** interact; however, no correlation was observed between their mRNA levels in TCGA database. Furthermore, qRT-PCR analysis revealed that ***TRIM17*** overexpression or silencing had no effect on ***FTO*** mRNA levels (Fig. 6A, B). However, Western blot results showed that after ***TRIM17*** overexpression, the level of ***FTO*** protein decreased significantly, suggesting that ***TRIM17*** may affect the expression of ***FTO*** protein through post-translational modification (Fig. 6C, D). Similarly, after silencing TRIM17, the expression level of FTO protein was significantly increased (Fig. 6E, F). To evaluate the stability of the ***FTO*** protein, the cells were treated with CHX (100 μg/mL) to inhibit the synthesis of new proteins, while actinomycin D (5 μg/mL) was used to inhibit mRNA transcription. Cells were collected at 0, 2, 4, and 8 h, respectively. The level of ***FTO*** protein was detected by Western blot. With ***GAPDH*** as the internal reference, the kinetics of protein degradation was analyzed. The results showed that overexpression of ***TRIM17*** significantly reduced the stability of ***FTO*** protein in 143B cells, whereas silencing ***TRIM17*** expression significantly enhanced ***FTO*** protein stability (Fig. 6G–J). The same results were observed in U2OS cells (Supplementary Fig. S3A–D). The results of in vivo ubiquitination assays demonstrated that overexpression of ***TRIM17*** promoted the ubiquitination of ***FTO*** protein, whereas silencing ***TRIM17*** inhibited ***FTO*** protein ubiquitination, indicating that ***TRIM17*** may regulate ***FTO*** expression through modulating its post-translational ubiquitination modification (Fig. 6K, L and Supplementary Fig. S3E, F). Subsequently, we investigated whether ***TRIM17*** influences tumor progression by modulating ***FTO*** protein expression and regulating the ***AKT/mTOR*** signaling pathway. Western blot analysis revealed that overexpression of ***FTO*** partially reversed the increased phosphorylation levels of **AKT,** ***mTOR***, and ***S6K1*** proteins caused by ***TRIM17*** overexpression (Fig. 6M–O). In contrast, silencing ***FTO*** expression partially reversed the reduction of ***AKT***, ***mTOR***, and ***S6K1*** protein phosphorylation levels caused by ***TRIM17*** knockdown (Supplementary Fig. S3G–I). The results of CCK-8, plate cloning, wound healing, and transwell invasion assays demonstrated that overexpression of ***FTO*** protein expression partially reversed or counteracted the effects of ***TRIM17*** overexpression on cell clonability and survival potential, migration, and invasion (Supplementary Fig. S4A–H). In conclusion, ***TRIM17*** regulates ***FTO*** expression by promoting post-translational ubiquitination modification of ***FTO*** protein, thereby positively modulating the ***AKT/mTOR*** signaling pathway to enhance the clonability and survival potential, migration, and invasion of osteosarcoma.

### TRIM17 regulates PDK1 through FTO-mediated m6A demethylation and activates AKT/mTOR signaling pathway activity

**Fig. 7:**
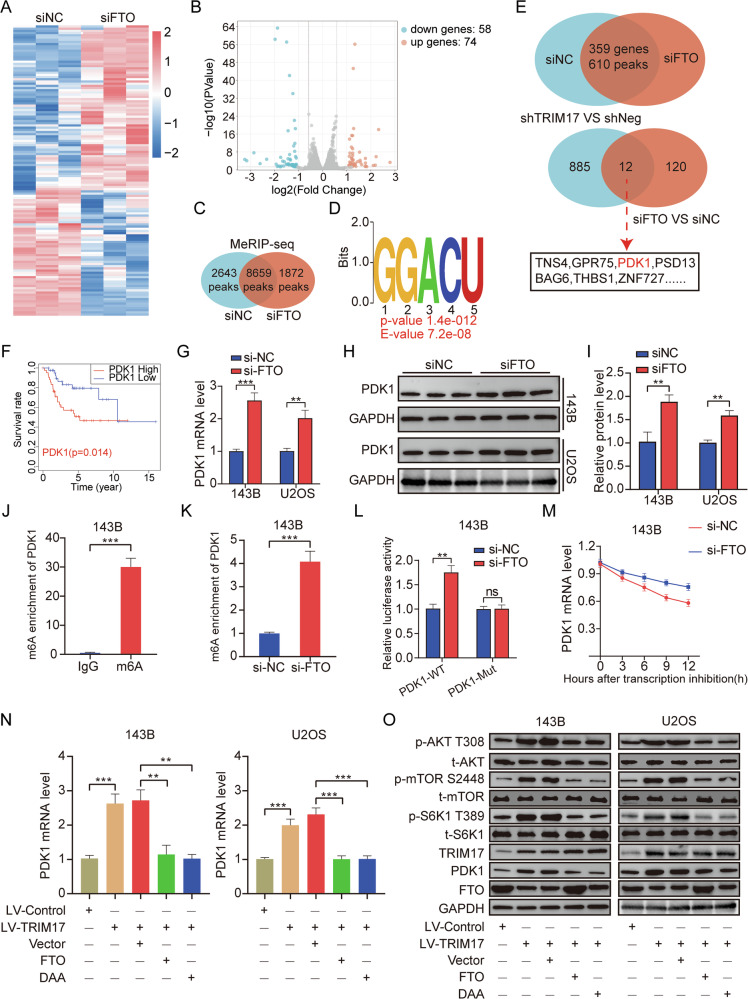
*FTO*-mediated m6A demethylation regulates *PDK1* mRNA stability. **A**, **B** Differential gene expression in si-***NC*** group and si-***FTO*** group. **C** The number of m6A enriched regions in MeRIP-seq sequencing. **D** m6A modifies the motif region. **E** Venn map of differential genes. **F** Kaplan–Meier survival analysis of ***PDK1*** gene. **G–I** The effect of ***FTO*** expression on ***PDK1*** expression level. **J** MeRIP assay verified the existence of m6A modification in ***PDK1***. **K** Effect of ***FTO*** on ***PDK1*** m6A modification level. **L** Influence of Dual-Luciferase Reporter Assay on plasmid luciferase activity. The ***PDK1-MUT*** is a c.301 A → G mutation (corresponding to p.Lys101 →Glu) in the PH domain. **M** Effect of ***FTO*** on the half-life of ***PDK1*** mRNA. **N** The levels of ***PDK1*** mRNA were detected by qRT-PCR. **O** Western blot was used to detect the protein expression in each group. Student’s *t*-test (two groups) and one-way analysis of variance (more than two groups) were used to analyze the differences between groups. All data are expressed as mean ± standard deviation (SD). ** *P* < 0.01, *** *P* < 0.001.

To further investigate the role of the ***FTO*** gene in osteosarcoma, we performed RNA-seq analysis on the control cell line (***siNC***) and the ***FTO*** knockdown cell line (***si-FTO***) in 143B cells. The results revealed that 132 genes were differentially expressed after ***FTO*** knockdown, with 58 genes downregulated and 74 genes upregulated (Fig. 7A, B). MeRIP-Seq analysis results demonstrated that, compared to the ***si-NC*** group, the ***si-FTO*** group exhibited 1872 specific m6A enrichment regions, with “GGACU” representing the most significantly enriched m6A modification motif (Fig. 7C, D). Based on the integrated analysis of RNA-Seq and MeRIP-Seq data, 359 differentially expressed genes following ***FTO*** knockdown contained m6A enrichment regions, and 12 overlapping genes, including ***TNS4***, ***GPR75***, and ***PDK1***, were identified through combined screening of previously identified genes and those differentially expressed due to ***TRIM17*** knockdown (Fig. 7E). Among them, 3-phosphoinositol-dependent protein kinase 1 (***PDK1***) is a key regulator of the AGC kinase family, regulating its activity through phosphorylation of the activation loop of downstream substrates, including ***AKT***, ***PKC***, and ***SGK***. The regulatory role of ***PDK1*** is particularly critical in the ***PI3K/AKT/mTOR*** signaling pathway [29]. Studies have demonstrated that the promoter region of the ***PDK1*** gene is regulated by DNA methylation, and ***PDK1*** methylation may interact with other post-translational modifications to coordinately regulate its function [30]. Kaplan–Meier survival analysis showed that high expression of ***PDK1*** was associated with shorter overall survival (Fig. 7F). Furthermore, univariate and multivariate COX analyses showed that ***PDK1*** was an independent prognostic factor in patients with osteosarcoma (Supplementary Fig. S5A, B). qRT-PCR and Western blot results showed that ***FTO*** knockdown could significantly increase ***PDK1*** mRNA and protein levels (Fig. 7G–I). MeRIP result showed that ***PDK1*** was modified by m6A (Fig. 7J and Supplementary Fig. S5C). MeRIP-qPCR results showed that ***FTO*** knockdown could significantly enhance the ***PDK1*** m6A modification level (Fig. 7K and Supplementary Fig. S5D). The results of the Dual-Luciferase Reporter Assay demonstrated that ***FTO*** knockdown significantly increased the luciferase activity of the ***PDK1-WT*** plasmid but did not affect the luciferase activity of the ***PDK1-Mut*** plasmid, suggesting that ***FTO*** inhibits the m6A modification of ***PDK1*** (Fig. 7L and Supplementary Fig. S5E). qRT-PCR results showed that ***FTO*** knockdown could significantly increase the half-life of ***PDK1*** mRNA, combined with the MeRIP-Seq results, it was confirmed that ***FTO*** regulates the stability of ***PDK1*** mRNA through m6A modification (Fig. 7M and Supplementary Fig. S5F). Next, we investigated whether ***TRIM17*** regulates ***PDK1*** via ***FTO***-mediated m6A demethylation and activates the ***AKT/mTOR*** signaling pathway. It was found by qRT-PCR detection that overexpression of ***TRIM17*** significantly increased the level of ***PDK1*** mRNA, while overexpression of ***FTO*** or the use of RNA methylation inhibitor DAA (2′-deoxyadenosine, Can inhibit the activity of m6A methyltransferase) could reverse the increase of ***PDK1*** mRNA induced by ***TRIM17*** (Fig. 7N). Western blot results showed that DAA treatment could inhibit the activity of m6A methyltransferase and reduce the m6A modification level of ***PDK1***, thereby reversing the activation of the ***AKT/mTOR*** pathway induced by ***TRIM17***, which was consistent with the effect of ***FTO*** overexpression (Fig. 7O and Supplementary Fig. S5G–H). Therefore, our assayal results suggest that ***TRIM17*** regulates the expression of ***PDK1*** through ***FTO***-mediated m6A demethylation in osteosarcoma, and further activates downstream ***AKT/mTOR*** signaling pathway activity to positively regulate the malignancy of osteosarcoma.

### Silencing the expression of TRIM17 can inhibit tumor growth in vivo

**Fig. 8:**
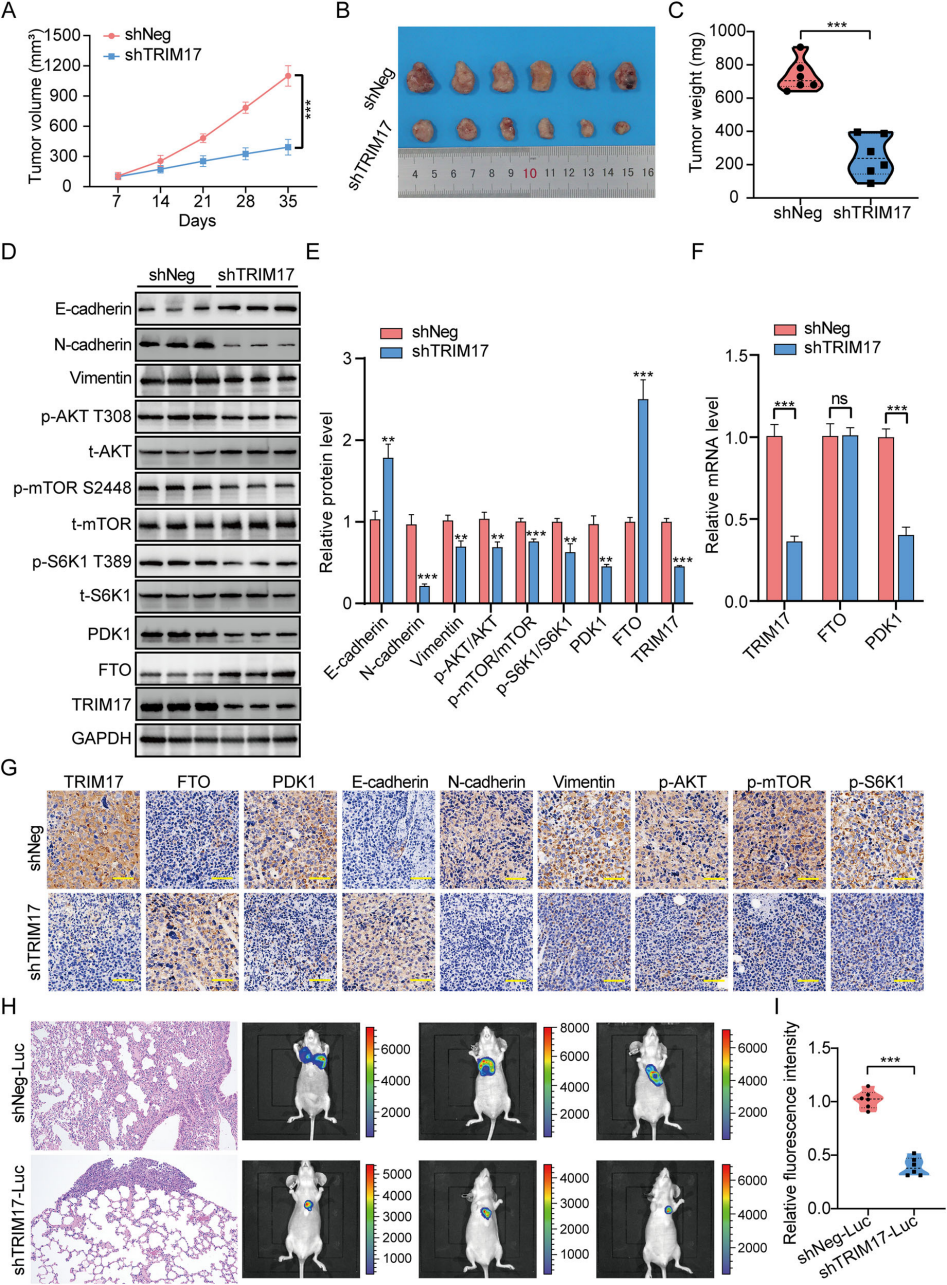
*TRIM17* influences the progression of osteosarcoma in vivo via the *FTO/PDK1/AKT/mTOR* signaling axis. **A–C** Silencing ***TRIM17*** can inhibit the volume and weight of osteosarcoma in vivo. **D, E** The expression levels of ***TRIM17, FTO, PDK1, EMT***-related proteins, and ***AKT/mTOR*** related proteins in subcutaneous tumor tissues were detected by western blot. **F** mRNA levels of ***TRIM17, FTO***, and ***PDK1*** in subcutaneous tumor tissues were detected by qRT-PCR. **G** The expression levels of ***TRIM17, FTO, PDK1***, ***EMT***-related proteins, and ***AKT/mTOR*** related proteins in subcutaneous tumor tissue samples were analyzed by immunohistochemistry (IHC). Scale: 400 μm. **H**, **I** HE staining and in vivo imaging showed the number of lung metastases in nude mice in the ***shNeg-Luc*** and ***shTRIM17-Luc*** groups. Scale: 200 μm. Student’s *t*-test (two groups) and one-way analysis of variance (more than two groups) were used to analyze the differences between groups. All data are expressed as mean ± standard deviation (SD). ** *P* < 0.01, *** *P* < 0.001.

We injected 143B osteosarcoma cells with stable ***TRIM17*** knockdown or control cells into the flanks of nude mice, monitored the tumor development of the mice, and measured tumor size every 7 days. The assayal results showed that the SC tumors of nude mice in the ***shTRIM17*** group were much smaller in volume and weight than those in the control group (Fig. 8A–C), suggesting that inhibiting ***TRIM17*** expression in vivo can suppress the growth of osteosarcoma. Subsequently, the tumor cells and tissues of each group were extracted for western blotting, qRT-PCR, and IHC assays (Fig. 8D–G). The assayal results demonstrated that the expression levels of ***p-AKT T308, p-mTOR S2448, p-S6K1 T389***, and ***FTO*** in the ***shTRIM17*** group were significantly reduced, whereas the expression level of ***PDK1*** protein was increased. Finally, we injected stably transfected osteosarcoma cells into mice via the tail vein to construct a lung metastasis model of osteosarcoma, through which we investigated the effect of silencing ***TRIM17*** on distant metastasis of osteosarcoma in vivo. Hematoxylin-eosin staining more intuitively showed that lung metastasis in the ***shTRIM17-Luc*** group was significantly reduced, and the luciferase activity of ***shTRIM17-Luc*** cells was also significantly lower than that of ***shNeg-Luc*** cells, which was consistent with the reduction of metastasis burden, suggesting that silencing ***TRIM17*** can inhibit lung metastasis of osteosarcoma (Fig. 8H, I). In conclusion, in vivo assays revealed that ***TRIM17*** positively regulates the activation of the ***AKT/mTOR*** signaling pathway via ***FTO***-mediated ***PDK1*** expression, thereby enhancing osteosarcoma growth.

## Discussion

Osteosarcoma is a common malignant primary bone tumor in adolescents and children [31]. It has a high degree of malignancy and is prone to metastasis in the early stage [5]. Although medical advances have significantly improved the survival rate of osteosarcoma patients, early screening and diagnosis remain challenging due to the absence of specific diagnostic markers. Therefore, elucidating the pathogenesis of osteosarcoma to improve patient prognosis is crucial for the early diagnosis and treatment of this disease.

***TRIM*** family proteins play an important role in protein post-translational modification systems [13]. Ubiquitination plays an important role in protein localization, metabolism, regulation, and degradation [32]. ***TRIM17*** is an E3 ubiquitin ligase belonging to the ***TRIM*** protein family. The ***TRIM17*** protein has a RING domain and two B-box domains [21]. ***TRIM17*** regulates the ubiquitination of target proteins through its E3 ubiquitin ligase activity, affecting the stability and function of these proteins.

As an E3 ubiquitin ligase, ***TRIM17*** promotes ***FTO*** ubiquitination and degradation, thereby activating the ***AKT/mTOR*** pathway to drive osteosarcoma progression. This mechanism aligns with its role in regulating target protein stability via the ubiquitin-proteasome system, as previously introduced. It is worth noting that this study reveals for the first time a new mechanism by which ***TRIM17*** activates the ***AKT/mTOR*** pathway by degrading ***FTO*** and enhancing the m6A modification of ***PDK1***. This is different from the pattern mentioned in the introduction that ***TRIM17*** promotes cell survival by degrading ***BAX*** in gastric cancer. It is suggested that ***TRIM17*** may exert a pro-cancer effect by targeting different substrates in different tumors. RNA sequencing analysis revealed that only ***TRIM17*** and ***TRIM26*** genes within the ***TRIM*** family were differentially expressed. In our previous studies, ***TRIM26*** was associated with osteosarcoma progression [14]. We further investigated the mechanism of action of ***TRIM17*** in osteosarcoma. Analysis of tissues and osteosarcoma cell lines from clinical patients revealed that ***TRIM17*** expression was significantly elevated in osteosarcoma, and high ***TRIM17*** expression correlated with poor prognosis in osteosarcoma patients. In vitro assays demonstrated that silencing ***TRIM17*** can inhibit the clonability and survival potential, migration, and invasion of osteosarcoma, whereas ***TRIM17*** overexpression promoted these malignant behaviors. Therefore, ***TRIM17*** functions as a potential oncogene in osteosarcoma, promoting tumor malignancy, and represents a potential therapeutic target for osteosarcoma patient.

To further elucidate the mechanism of ***TRIM17*** in osteosarcoma progression, RNA-Seq analysis was performed on the ***TRIM17*** knockdown group and the control group, revealing that ***TRIM17*** primarily regulates the ***AKT/mTOR*** signaling pathway. The ***AKT/mTOR*** signaling pathway is a critical regulatory network in cell biology, integrating signals from nutrients, energy, and growth factors [7, 33]. Abnormal activation of this pathway is strongly associated with multiple types of cancer and has become a key target in cancer research and treatment [34–36]. Activation of the ***AKT/mTOR*** signaling pathway is strongly associated with the clonability and survival potential and metastasis of breast cancer cells [37, 38]. Abnormal activation of this pathway is associated with the occurrence and progression of colorectal cancer, affecting tumor aggressiveness and drug resistance [39–41]. In hepatocellular carcinoma, activation of the ***AKT/mTOR*** signaling pathway promotes tumor cell clonability and survival potential and metabolic alterations, leading to poor prognosis [42, 43]. Therefore, we investigated the changes in ***AKT/mTOR*** signaling pathway-related proteins in the ***shTRIM17*** group and ***LV-TRIM17*** groups, respectively. The results demonstrated that silencing ***TRIM17*** inhibited the activation of the ***AKT/mTOR*** signaling pathway activation, whereas ***TRIM17*** overexpression enhanced its activation. In conclusion, ***TRIM17*** likely modulates osteosarcoma malignancy through regulation of the ***AKT/mTOR*** signaling pathway.

Subsequently, through mass spectrometry and molecular docking assays, we identified that ***TRIM17*** regulates the ***AKT/mTOR*** signaling pathway by inducing ***FTO*** protein degradation. ***FTO*** is a protein with demethylase activity, primarily functioning to remove methyl modifications, particularly m6A, from RNA and DNA [44]. m6A is the most common RNA methylation modification, affecting RNA stability, transport, and translation [10]. Recent studies have demonstrated that ***FTO*** plays a critical role in the pathogenesis and progression of various cancers. In breast cancer, high ***FTO*** expression is linked to tumor malignancy and poor patient prognosis [23]. The upregulation of ***FTO*** in hepatocellular cancer cells promotes cell clonability and survival potential and migration, which may be related to the alteration of m6A methylation [45]. Overexpression of ***FTO*** is also thought to be associated with increased clonability and survival potential and aggressiveness of gastric cancer cells [46]. This study demonstrated that ***TRIM17*** interacts with ***FTO*** and induces its ubiquitination-mediated degradation. In vitro rescue assays revealed that ***FTO*** overexpression partially reversed ***TRIM17***-induced activation of the ***AKT/mTOR*** signaling pathway and attenuated osteosarcoma malignancy. It is worth emphasizing that the conclusion in this study that ***TRIM17*** promotes the progression of osteosarcoma by activating the ***mTOR*** pathway is highly consistent with previous studies. For instance, Adewuyi et al. discovered that the activation of the ***mTOR*** pathway is a key mechanism for osteosarcoma cells to evade chemotherapy [4]; Zheng et al. confirmed that the abnormal activation of the ***PDK1-AKT-mTOR*** axis could drive the invasive phenotype of osteosarcoma cells [29]. These findings jointly support the importance of the ***mTOR*** pathway as a therapeutic target for osteosarcoma and also provide a theoretical basis for ***TRIM17*** as a new therapeutic target. In conclusion, ***TRIM17*** promotes ***FTO*** degradation via ubiquitination, a post-translational modification, and positively modulates the ***AKT/mTOR*** signaling pathway, thereby enhancing osteosarcoma malignancy.

To determine whether ***FTO*** also functions as a demethylase in osteosarcoma progression, we performed RNA-seq and MeRIP-Seq analyses in the ***FTO*** knockdown group and the control group. The results revealed specific m6A enrichment regions in the ***si-FTO*** group. Integrating differential gene analysis following ***TRIM17*** knockdown, we found that ***FTO*** likely regulates ***PDK1*** protein through modulation of its methylation levels. The results demonstrated that ***PDK1*** contains m6A modifications, and ***FTO*** knockdown significantly elevated the m6A modification levels of ***PDK1***. Further analysis revealed that ***FTO*** overexpression or treatment with the methylation inhibitor DAA reversed the ***TRIM17***-induced increase in ***PDK1*** mRNA levels and the phosphorylation levels of ***AKT T308, mTOR S2448,*** and ***p-S6K1 T389***. It is worth noting that ***TRIM17***, as an E3 ubiquitin ligase, shows a high substrate specificity in the degradation of ***FTO***. This process is inhibited by releasing the m6A modification of ***PDK1***, forming the ***Trim17-FTO-PDK1*** signaling axis. This regulatory pattern is different from the mechanism by which ***TRIM17*** inhibits apoptosis by degrading ***BAX*** in gastric cancer [17], suggesting that ***TRIM17*** may exert a pro-cancer effect by targeting specific substrates in different tumors. In this study, the anti-cancer effect of ***FTO*** is consistent with its function as an m6A demethylase—low expression of ***FTO*** enhances the m6A modification of ***PDK1*** mRNA, thereby stabilizing ***PDK1*** and activating the pro-cancer ***AKT/mTOR*** pathway. This is not contradictory to the conclusion in previous studies that ***FTO*** promotes cancer through the Wnt pathway [47]. The controversy over the expression of ***FTO*** in osteosarcoma essentially stems from its pleiotropy as an m6A demethylase—when ***FTO*** targets Wnt pathway-related genes (such as ***DACT1***), high expression promotes tumor progression; when ***FTO*** targets ***AKT/mTOR*** pathway genes (such as ***PDK1***), low expression activates pro-cancer signals by enhancing m6A modification. This phenomenon suggests that the function of ***FTO*** is “target gene-dependent”, and its ultimate biological effect depends on the activation status of the signaling pathways in the cellular microenvironment. In summary, ***TRIM17*** binds to ***FTO***, promoting its ubiquitination and degradation, enhances ***PDK1*** mRNA stability in an m6A-dependent manner, activates the ***AKT/mTOR*** signaling axis, and drives osteosarcoma cell clonability and survival potential and invasion, thereby contributing to osteosarcoma progression.

However, our study has several limitations. First, the clinical sample size is limited: the small number of clinical samples may introduce bias in the statistical analysis and compromise the generalizability and reliability of the findings. Second, the mechanistic studies lack depth: the interaction between ***TRIM17*** and ***FTO*** has not been fully elucidated, including the specific ubiquitination sites and types of ***FTO***, as well as the mechanism by which ***TRIM17*** recognizes ***FTO***. The specific m6A modification sites on ***PDK1*** mRNA and their effects on ***PDK1*** translation and degradation remain to be investigated in detail. Third, the in vivo assays have limitations: the study relied solely on a nude mouse SC xenograft model and an in vivo metastatic model to validate ***TRIM17*** function in vivo, which does not fully recapitulate the human osteosarcoma microenvironment. These limitations will be addressed and further explored in future studies.

In conclusion, we have elucidated the mechanism by which ***TRIM17*** positively regulates osteosarcoma progression. Elevated ***TRIM17*** expression is associated with poor prognosis in osteosarcoma patients. Mechanistically, ***TRIM17*** regulates ***FTO*** degradation via ubiquitination, modulates ***PDK1*** gene methylation, and thereby activates the ***AKT/mTOR*** signaling pathway, promoting osteosarcoma malignancy. Future studies should focus on a deeper exploration of ***TRIM17’s*** regulatory mechanisms in osteosarcoma and its interactions with other signaling pathways to develop more effective therapeutic strategies. ***TRIM17*** represents a novel therapeutic target for osteosarcoma, and its combination with other targeted therapies or immunotherapies is expected to enhance treatment efficacy and improve patient outcomes.

## Supplementary information

Supplementary Figure S1

Supplementary Figure S2

Supplementary Figure S3

Supplementary Figure S4

Supplementary Figure S5

Supplementary Figure legends

Supplementary table

Original blot

## Data Availability

Differential gene analysis is performed using the DESeq2 package, KEGG enrichment analysis employs the clusterProfiler package, and m6A-enriched regions identification utilizes the MeRIPseqR package. All of these tools are publicly available standardized analysis packages, without any custom code.

## References

[1] Yu S, Yao X. Advances on immunotherapy for osteosarcoma. Mol Cancer. 2024;23:192. 39245737 10.1186/s12943-024-02105-9 PMC11382402

[2] Tian H, Cao J, Li B, Nice EC, Mao H, Zhang Y, et al. Managing the immune microenvironment of osteosarcoma: the outlook for osteosarcoma treatment. Bone Res. 2023;11:11. 36849442 10.1038/s41413-023-00246-z PMC9971189

[3] Zhang W, Li L, Wang Z, Nie Y, Yang Y, Li C, et al. Injectable and adhesive MgO(2)-potentiated hydrogel with sequential tumor synergistic therapy and osteogenesis for challenging postsurgical osteosarcoma treatment. Biomaterials. 2025;315:122959. 39612764 10.1016/j.biomaterials.2024.122959

[4] Adewuyi E, Chorya H, Muili A, Moradeyo A, Kayode A, Naik A. et al. Chemotherapy, immunotherapy, and targeted therapy for osteosarcoma: recent advancements. Crit Rev Oncol Hemat. 2025;206:104575 10.1016/j.critrevonc.2024.104575 39581243

[5] Beird HC, Bielack SS, Flanagan AM, Gill J, Heymann D, Janeway KA, et al. Osteosarcoma. Nat Rev Dis Prim. 2022;8:77. 36481668 10.1038/s41572-022-00409-y

[6] O’Donnell JS, Massi D, Teng MWL, Mandala M. PI3K-AKT-mTOR inhibition in cancer immunotherapy, redux. Semin Cancer Biol. 2018;48:91–103. 28467889 10.1016/j.semcancer.2017.04.015

[7] Yu L, Wei J, Liu P. Attacking the PI3K/Akt/mTOR signaling pathway for targeted therapeutic treatment in human cancer. Semin Cancer Biol. 2022;85:69–94. 34175443 10.1016/j.semcancer.2021.06.019

[8] Gao X, Li J, Feng X, Xie Y, Zhang J, Liu J, et al. EHD1 promotes breast cancer metastasis through upregulating HIF2a expression via activating mTOR pathway. FASEB J. 2024;38:e70168. 39530565 10.1096/fj.202401919R

[9] Ren J, Hu Z, Niu G, Xia J, Wang X, Hong R, et al. Annexin A1 induces oxaliplatin resistance of gastric cancer through autophagy by targeting PI3K/AKT/mTOR. FASEB J. 2023;37:e22790. 36786694 10.1096/fj.202200400RR

[10] Chen C, Guo Y, Huang Q, Wang B, Wang W, Niu J. et al. PI3K inhibitor impairs tumor progression and enhances sensitivity to anlotinib in anlotinib-resistant osteosarcoma. Cancer Lett. 2022;536:215660 35318116 10.1016/j.canlet.2022.215660

[11] Basu-Shrivastava M, Kozoriz A, Desagher S, Lassot I. To ubiquitinate or not to ubiquitinate: TRIM17 in cell life and death. Cells. 2021;10:1235. 34069831 10.3390/cells10051235 PMC8157266

[12] Zhang L, Afolabi LO, Wan X, Li Y, Chen L. Emerging roles of tripartite motif-containing family proteins (TRIMs) in eliminating misfolded proteins. Front Cell Dev Biol. 2020;8:802. 32984318 10.3389/fcell.2020.00802 PMC7479839

[13] Cai C, Tang YD, Zhai J, Zheng C. The RING finger protein family in health and disease. Signal Transduct Target Ther. 2022;7:300. 36042206 10.1038/s41392-022-01152-2 PMC9424811

[14] Xia K, Zheng D, Wei Z, Liu W, Guo W. TRIM26 inhibited osteosarcoma progression through destabilizing RACK1 and thus inactivation of MEK/ERK signaling. Cell Death Dis. 2023;14:529. 37591850 10.1038/s41419-023-06048-9 PMC10435491

[15] Zheng D, Ning J, Deng H, Ruan Y, Cheng F. TRIM26 inhibits clear cell renal cell carcinoma progression through destabilizing ETK and thus inactivation of AKT/mTOR signaling. J Transl Med. 2024;22:481. 38773612 10.1186/s12967-024-05273-w PMC11110379

[16] Li L, Zhang Y, Hu W, Zou F, Ning J, Rao T, et al. MTHFD2 promotes PD-L1 expression via activation of the JAK/STAT signalling pathway in bladder cancer. J Cell Mol Med. 2023;27:2922–36. 37480214 10.1111/jcmm.17863 PMC10538262

[17] Shen JJ, Yang H, Qiao XR, Chen Y, Zheng LY, Lin JY, et al. The E3 ubiquitin ligase TRIM17 promotes gastric cancer survival and progression via controlling BAX stability and antagonizing apoptosis. Cell Death Differ. 2023;30:2322–35. 37697039 10.1038/s41418-023-01221-1 PMC10589321

[18] Zhong T, Zhang J, Liu X, Li H. TRIM17-mediated ubiquitination and degradation of RBM38 promotes cisplatin resistance in non-small cell lung cancer. Cell Oncol (Dordr). 2023;46:1493–507. 37219768 10.1007/s13402-023-00825-6 PMC12974751

[19] Lassot I, Mora S, Lesage S, Zieba BA, Coque E, Condroyer C, et al. The E3 ubiquitin ligases TRIM17 and TRIM41 modulate alpha-synuclein expression by regulating ZSCAN21. Cell Rep. 2018;25:2484–96.e2489. 30485814 10.1016/j.celrep.2018.11.002

[20] Lionnard L, Duc P, Brennan MS, Kueh AJ, Pal M, Guardia F, et al. TRIM17 and TRIM28 antagonistically regulate the ubiquitination and anti-apoptotic activity of BCL2A1. Cell Death Differ. 2019;26:902–17. 30042493 10.1038/s41418-018-0169-5 PMC6461866

[21] Magiera MM, Mora S, Mojsa B, Robbins I, Lassot I, Desagher S. Trim17-mediated ubiquitination and degradation of Mcl-1 initiate apoptosis in neurons. Cell Death Differ. 2013;20:281–92. 22976837 10.1038/cdd.2012.124 PMC3554334

[22] Chen A, Zhang VX, Zhang Q, Sze KM, Tian L, Huang H, et al. Targeting the oncogenic m6A demethylase FTO suppresses tumourigenesis and potentiates immune response in hepatocellular carcinoma. Gut. 2024;74:90–102. 38839271 10.1136/gutjnl-2024-331903 PMC11672076

[23] Niu Y, Lin Z, Wan A, Chen H, Liang H, Sun L, et al. RNA N6-methyladenosine demethylase FTO promotes breast tumor progression through inhibiting BNIP3. Mol Cancer. 2019;18:46. 30922314 10.1186/s12943-019-1004-4 PMC6437932

[24] Zhao T, Sun D, Xiong W, Man J, Zhang Q, Zhao M, et al. N(6)-methyladenosine plays a dual role in arsenic carcinogenesis by temporal-specific control of core target AKT1. J Hazard Mater. 2023;445:130468. 36444808 10.1016/j.jhazmat.2022.130468

[25] An X, Wang R, Lv Z, Wu W, Sun Z, Wu R, et al. WTAP-mediated m(6)A modification of FRZB triggers the inflammatory response via the Wnt signaling pathway in osteoarthritis. Exp Mol Med. 2024;56:156–67. 38172596 10.1038/s12276-023-01135-5 PMC10834961

[26] Huang J, Sun W, Wang Z, Lv C, Zhang T, Zhang D. et al. FTO suppresses glycolysis and growth of papillary thyroid cancer via decreasing stability of APOE mRNA in an N6-methyladenosine-dependent manner. J Exp Clin Cancer Res. 2022;41:42 35090515 10.1186/s13046-022-02254-z PMC8796435

[27] Liu WD, Xia KZ, Huang XH, Wei Z, Wei ZC, Wang XY. et al. HMGCL activates autophagy in osteosarcoma through á-HB mediated inhibition of the PI3K/AKT/mTOR signaling pathway. J Transl Med. 2025;23:219. 39985081 10.1186/s12967-025-06227-6 PMC11846287

[28] Yuan BS, Shi KX, Zha JM, Cai YJ, Gu Y, Huang K, et al. Nuclear receptor modulators inhibit osteosarcoma cell proliferation and tumour growth by regulating the mTOR signaling pathway (vol 14, 51, 2023). Cell Death Dis. 2025;16:51. 10.1038/s41419-022-05545-7 PMC9867777 36681687

[29] Zheng NN, Wei JQ, Wu DP, Xu Y, Guo JP. Master kinase PDK1 in tumorigenesis. Bba-Rev Cancer 2023;1878:188971. 10.1016/j.bbcan.2023.188971 37640147

[30] Wang S, Zhang X, Chen Q, Wu H, Cao S, Zhao S, et al. FTO activates PD-L1 promotes immunosuppression in breast cancer via the m6A/YTHDF3/PDK1 axis under hypoxic conditions. J Adv Res. 2024.10.1016/j.jare.2024.12.02639701379

[31] Ritter J, Bielack SS. Osteosarcoma. Ann Oncol. 2010;21:vii320–325. 20943636 10.1093/annonc/mdq276

[32] Popovic D, Vucic D, Dikic I. Ubiquitination in disease pathogenesis and treatment. Nat Med. 2014;20:1242–53. 25375928 10.1038/nm.3739

[33] Tewari D, Patni P, Bishayee A, Sah AN, Bishayee A. Natural products targeting the PI3K-Akt-mTOR signaling pathway in cancer: a novel therapeutic strategy. Semin Cancer Biol. 2022;80:1–17. 31866476 10.1016/j.semcancer.2019.12.008

[34] Ediriweera MK, Tennekoon KH, Samarakoon SR. Role of the PI3K/AKT/mTOR signaling pathway in ovarian cancer: biological and therapeutic significance. Semin Cancer Biol. 2019;59:147–60. 31128298 10.1016/j.semcancer.2019.05.012

[35] Leiphrakpam PD, Are C. PI3K/Akt/mTOR signaling pathway as a target for colorectal cancer treatment. Int J Mol Sci. 2024;25:3178. 38542151 10.3390/ijms25063178 PMC10970097

[36] Nepstad I, Hatfield KJ, Gronningsaeter IS, Reikvam H. The PI3K-Akt-mTOR signaling pathway in human acute myeloid leukemia (AML) cells. Int J Mol Sci. 2020;21:2907. 10.3390/ijms21082907 PMC7215987 32326335

[37] Nunnery SE, Mayer IA. Targeting the PI3K/AKT/mTOR pathway in hormone-positive breast cancer. Drugs. 2020;80:1685–97. 32894420 10.1007/s40265-020-01394-w PMC7572750

[38] Wu HT, Lin J, Liu YE, Chen HF, Hsu KW, Lin SH, et al. Luteolin suppresses androgen receptor-positive triple-negative breast cancer cell proliferation and metastasis by epigenetic regulation of MMP9 expression via the AKT/mTOR signaling pathway. Phytomedicine. 2021;81:153437. 33352494 10.1016/j.phymed.2020.153437

[39] Duan S, Huang W, Liu X, Liu X, Chen N, Xu Q, et al. IMPDH2 promotes colorectal cancer progression through activation of the PI3K/AKT/mTOR and PI3K/AKT/FOXO1 signaling pathways. J Exp Clin Cancer Res. 2018;37:304. 30518405 10.1186/s13046-018-0980-3 PMC6282329

[40] Weng ML, Chen WK, Chen XY, Lu H, Sun ZR, Yu Q, et al. Fasting inhibits aerobic glycolysis and proliferation in colorectal cancer via the Fdft1-mediated AKT/mTOR/HIF1alpha pathway suppression. Nat Commun. 2020;11:1869. 32313017 10.1038/s41467-020-15795-8 PMC7170903

[41] Pradeepa SV, Senapati S, Chakraborty S. AKT inhibition sensitizes EVI1 expressing colon cancer cells to irinotecan therapy by regulating the Akt/mTOR axis. Cell Oncol (Dordr). 2022;45:659–75. 35834097 10.1007/s13402-022-00690-9 PMC12978111

[42] Tian LY, Smit DJ, Jucker M. The role of PI3K/AKT/mTOR signaling in hepatocellular carcinoma metabolism. Int J Mol Sci. 2023;24:2652. 10.3390/ijms24032652 PMC9916527 36768977

[43] Zhang M, Liu S, Chua MS, Li H, Luo D, Wang S, et al. SOCS5 inhibition induces autophagy to impair metastasis in hepatocellular carcinoma cells via the PI3K/Akt/mTOR pathway. Cell Death Dis. 2019;10:612. 31406106 10.1038/s41419-019-1856-y PMC6690952

[44] Jiang X, Liu B, Nie Z, Duan L, Xiong Q, Jin Z, et al. The role of m6A modification in the biological functions and diseases. Signal Transduct Target Ther. 2021;6:74. 33611339 10.1038/s41392-020-00450-x PMC7897327

[45] Liu L, Gu M, Ma J, Wang Y, Li M, Wang H, et al. CircGPR137B/miR-4739/FTO feedback loop suppresses tumorigenesis and metastasis of hepatocellular carcinoma. Mol Cancer. 2022;21:149. 35858900 10.1186/s12943-022-01619-4 PMC9297645

[46] Xu YY, Li T, Shen A, Bao XQ, Lin JF, Guo LZ, et al. FTO up-regulation induced by MYC suppresses tumour progression in Epstein-Barr virus-associated gastric cancer. Clin Transl Med. 2023;13:e1505. 38082402 10.1002/ctm2.1505 PMC10713874

[47] Lv DM, Ding SR, Zhong L, Tu J, Li HB, Yao H, et al. M6A demethylase FTO-mediated downregulation of DACT1 mRNA stability promotes Wnt signaling to facilitate osteosarcoma progression. Oncogene. 2022;41:1727–41. 35121825 10.1038/s41388-022-02214-z

