# Supplementary figures and images for: TRIM17 promotes the progression of osteosarcoma by regulating PDK1 m6A modification-mediated AKT/mTOR pathway activation through ubiquitination of FTO
Source: Cell Death Dis. 2025 Oct 27;16(1):767. doi: 10.1038/s41419-025-08070-5 (PMC12559362; doi:10.1038/s41419-025-08070-5)

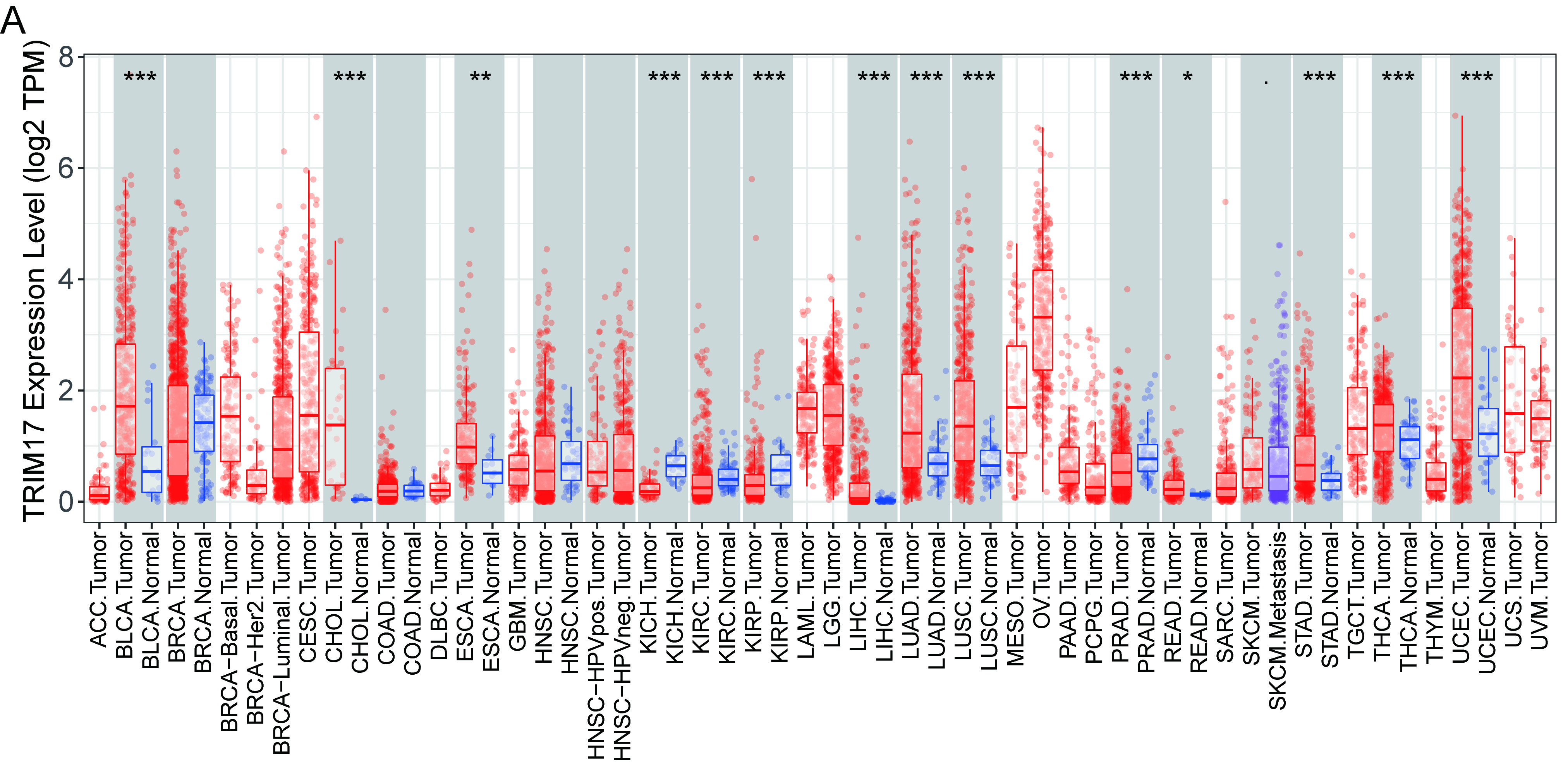

Supplement: Supplementary file 1 — Supplementary Figure S1 [file 41419_2025_8070_MOESM1_ESM.tif]
