# Supplementary material for: TRIM17 promotes the progression of osteosarcoma by regulating PDK1 m6A modification-mediated AKT/mTOR pathway activation through ubiquitination of FTO
Source: Cell Death Dis. 2025 Oct 27;16(1):767. doi: 10.1038/s41419-025-08070-5 (PMC12559362; doi:10.1038/s41419-025-08070-5)
Supplement: Supplementary file 6 — Supplementary Figure legends [file 41419_2025_8070_MOESM6_ESM.docx]

**Supplementary Figure S1| (A)** Analysis of ***TRIM17*** expression between multiple tumors and normal tissues.

**Supplementary Figure S2 |** Overexpression of ***TRIM17*** promotes clonability and survival potential, migration and invasion of osteosarcoma cells. **(A-C)** The expression level of ***TRIM17*** in overexpressed stable cells was verified. **(D-G)** The effect of overexpression of ***TRIM17*** on cell clonability and survival potential was detected by CCK-8 and colony formation assay, scale bar: 200 μm. **(H-I)** Transwell invasion assay was used to detect the effect of overexpression of ***TRIM17*** on cell invasion ability, scale bar: 200 μm. **(J-K)** The effect of overexpression of ***TRIM17*** on cell migration was detected by wound healing assay, scale bar: 200 μm. **(J-L)** Effect of overexpression of ***TRIM17*** on expression of EMT-related proteins. Student's t-test (two groups) and one-way analysis of variance (more than two groups) were used to analyze the differences between groups. All data are expressed as mean ± standard deviation (SD). ** P<0.01, *** P <0.001.

**Supplementary Figure S3| *TRIM17*** promotes ubiquitination and degradation of ***FTO*** protein. **(A-D)** The effect of ***TRIM17*** on the stability of ***FTO*** protein was detected by actinomycoketone assay. **(E-F)** Effect of ***TRIM17*** on ubiquitination level of ***FTO*** protein. **(G-I)** Silenced ***FTO*** regulates the effects of silenced ***TRIM17*** expression on the ***AKT/mTOR*** signaling pathway. Student's t-test (two groups) and one-way analysis of variance (more than two groups) were used to analyze the differences between groups. All data are expressed as mean ± standard deviation (SD). ** P <0.01, *** P <0.001.

**Supplementary Figure S4|** The overexpression of ***FTO*** partially reversed the effect of ***TRIM17*** overexpression on the malignancy of osteosarcoma. **(A-D)** The effects of overexpression of ***FTO*** on overexpression of ***TRIM17*** on cell proliferation, clonability and survival potential were verified by CCK-8 and plate cloning assays. **(E-G)** The effect of overexpression of ***FTO*** on overexpression of ***TRIM17*** on cell proliferation was verified by wound healing assay, scale bar: 200 μm. **(H-I)** The effect of overexpression of ***FTO*** on overexpression of ***TRIM17*** on cell proliferation was verified by Transwell invasion assay, scale bar: 200 μm. Student's t-test (two groups) and one-way analysis of variance (more than two groups) were used to analyze the differences between groups. All data are expressed as mean ± standard deviation (SD). * P <0.05, ** P <0.01, *** P <0.001.

**Supplementary Figure S5| *FTO***-mediated m6A methylation regulates ***PDK1*** mRNA stability. **(A-B)** Univariate and multifactorial COX analysis of ***PDK1*** gene. **(C)** MeRIP assay verified the existence of m6A modification in ***PDK1***. **(D)** Effect of ***FTO*** on ***PDK1*** m6A modification level. **(E)** Influence of luciferase reporter gene assay on plasmid luciferase activity. **(F)** Effect of ***FTO*** on the half-life of ***PDK1*** mRNA. **(G-H)** Quantitative analysis of protein expression in each group. Student's t-test (two groups) and one-way analysis of variance (more than two groups) were used to analyze the differences between groups. All data are expressed as mean ± standard deviation (SD). ** P <0.01, *** P <0.001.
