# Supplementary material for: TRIM17 promotes the progression of osteosarcoma by regulating PDK1 m6A modification-mediated AKT/mTOR pathway activation through ubiquitination of FTO
Source: Cell Death Dis. 2025 Oct 27;16(1):767. doi: 10.1038/s41419-025-08070-5 (PMC12559362; doi:10.1038/s41419-025-08070-5)
Supplement: Supplementary file 7 — Supplementary table [file 41419_2025_8070_MOESM7_ESM.docx]

Supplementary Table 1. Details of the antibodies

| Antibodies | Company | Catalog number | Species | WB Dilution | IHC Dilution | Co-IP Dilution |  |
| --- | --- | --- | --- | --- | --- | --- | --- |
| TRIM17 | Abcam | ab235527 | Rabbit | 1:1000 | 1:200 | 5 μg/800 μL lysate |  |
| GAPDH | ABclonal | AC001 | Rabbit | 1:10000 | 1:5000 |  |  |
| E-cadherin | ABclonal | A20798 | Rabbit | 1:1000 | 1:200 |  |  |
| N-cadherin | ABclonal | A0433 | Rabbit | 1:500 | 1:100 |  |  |
| Vimentin | ABclonal | A19607 | Rabbit | 1:20000 | 1:5000 |  |  |
| p-AKT T308 | ABclonal | AP1332 | Rabbit | 1:1000 | 1:200 |  |  |
| t-AKT | Servicebio | GB15689 | Rabbit | 1:1000 | 1:200 |  |  |
| p-mTOR s2448 | ABclonal | AP0115 | Rabbit | 1:1000 | 1:200 |  |  |
| t-mTOR | Servicebio | GB11405 | Rabbit | 1:1000 | 1:200 |  |  |
| p-S6K1 T389 | ABclonal | AP1389 | Rabbit | 1:2000 | 1:400 |  |  |
| t-S6K1 | Servicebio | GB111133 | Rabbit | 1:1000 | 1:200 |  |  |
| FTO | Abcam | ab126605 | Rabbit | 1:10000 | 1:2000 | 5 μg/800 μL lysate |  |
| Flag | Servicebio | GB15939 | Rabbit | 1:2000 | 1:400 | 5 μg/800 μL lysate |  |
| HA | Servicebio | GB151253 | Rabbit | 1:1000 | 1:200 | 5 μg/800 μL lysate |  |
| PDK1 | ABclonal | A8930 | Rabbit | 1:10000 | 1:2000 |  |  |
