# Supplementary material for: TRIM17 promotes the progression of osteosarcoma by regulating PDK1 m6A modification-mediated AKT/mTOR pathway activation through ubiquitination of FTO
Source: Cell Death Dis. 2025 Oct 27;16(1):767. doi: 10.1038/s41419-025-08070-5 (PMC12559362; doi:10.1038/s41419-025-08070-5)

Figure1

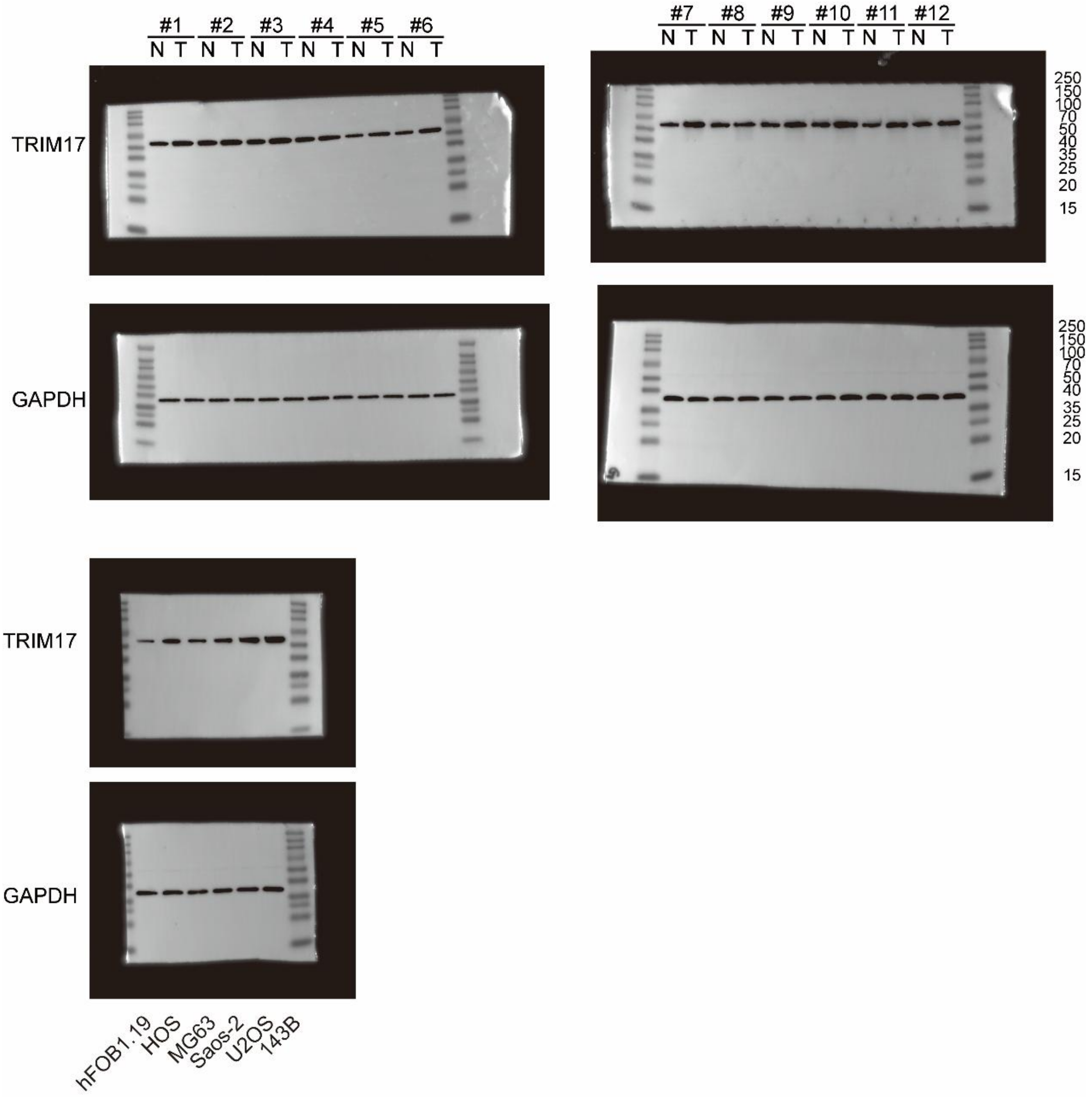

#1 #2 #3 #4 #5 #6
N T N T N T N T N T N T
#7 #8 #9 #10 #11 #12
N T N T N T N T N T N T
TRIM17
GAPDH
250
150
100
70
50
40
35
25
20
15
TRIM17
GAPDH
hFOB1.19
HOS
MG63
Saos-2
U2OS
143B

Figure2

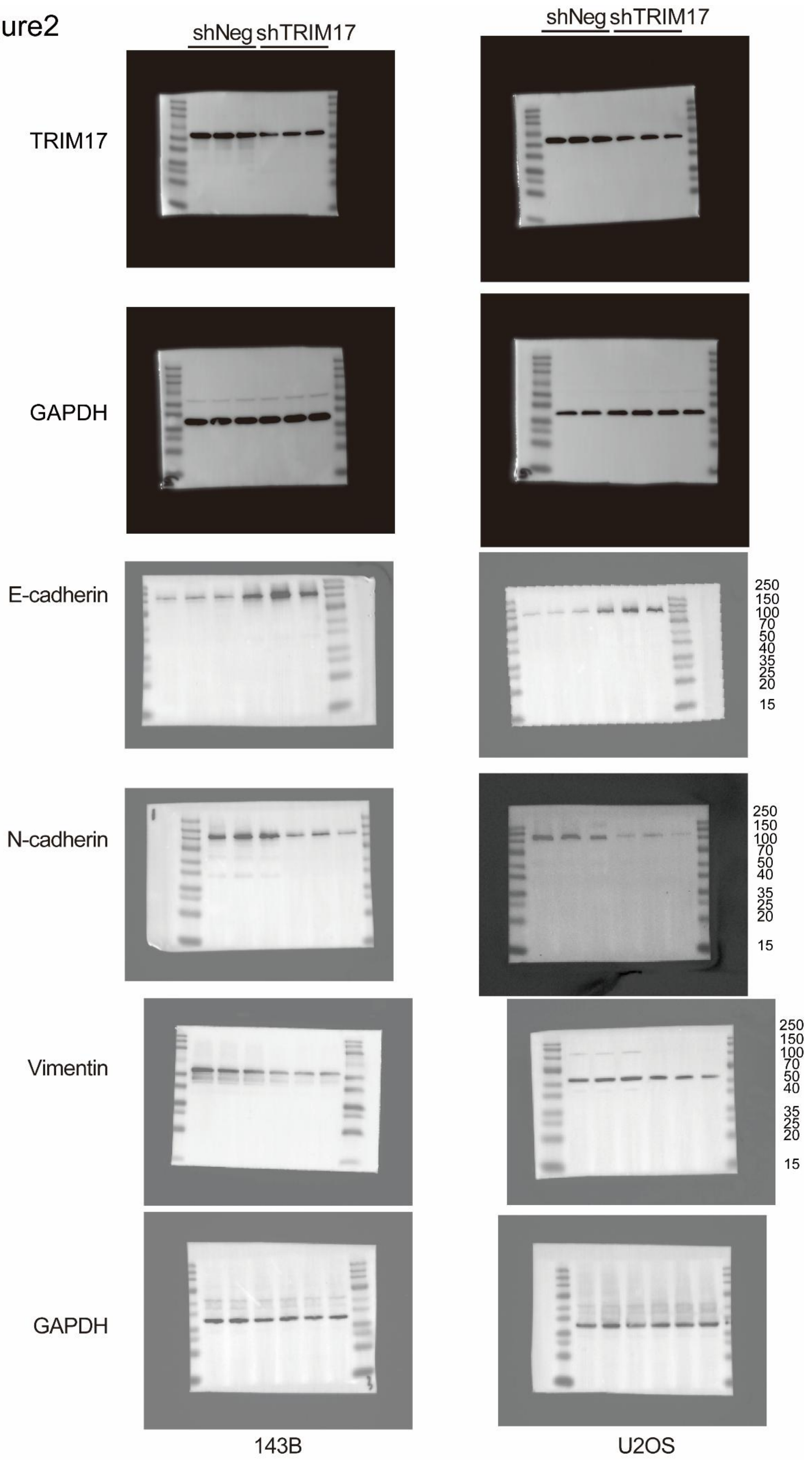

shNeg shTRIM17
shNeg shTRIM17
TRIM17
GAPDH
E-cadherin
250
150
100
70
50
40
35
25
20
15
N-cadherin
250
150
100
70
50
40
35
25
20
15
Vimentin
250
150
100
70
50
40
35
25
20
15
GAPDH
143B
U2OS

Figure3

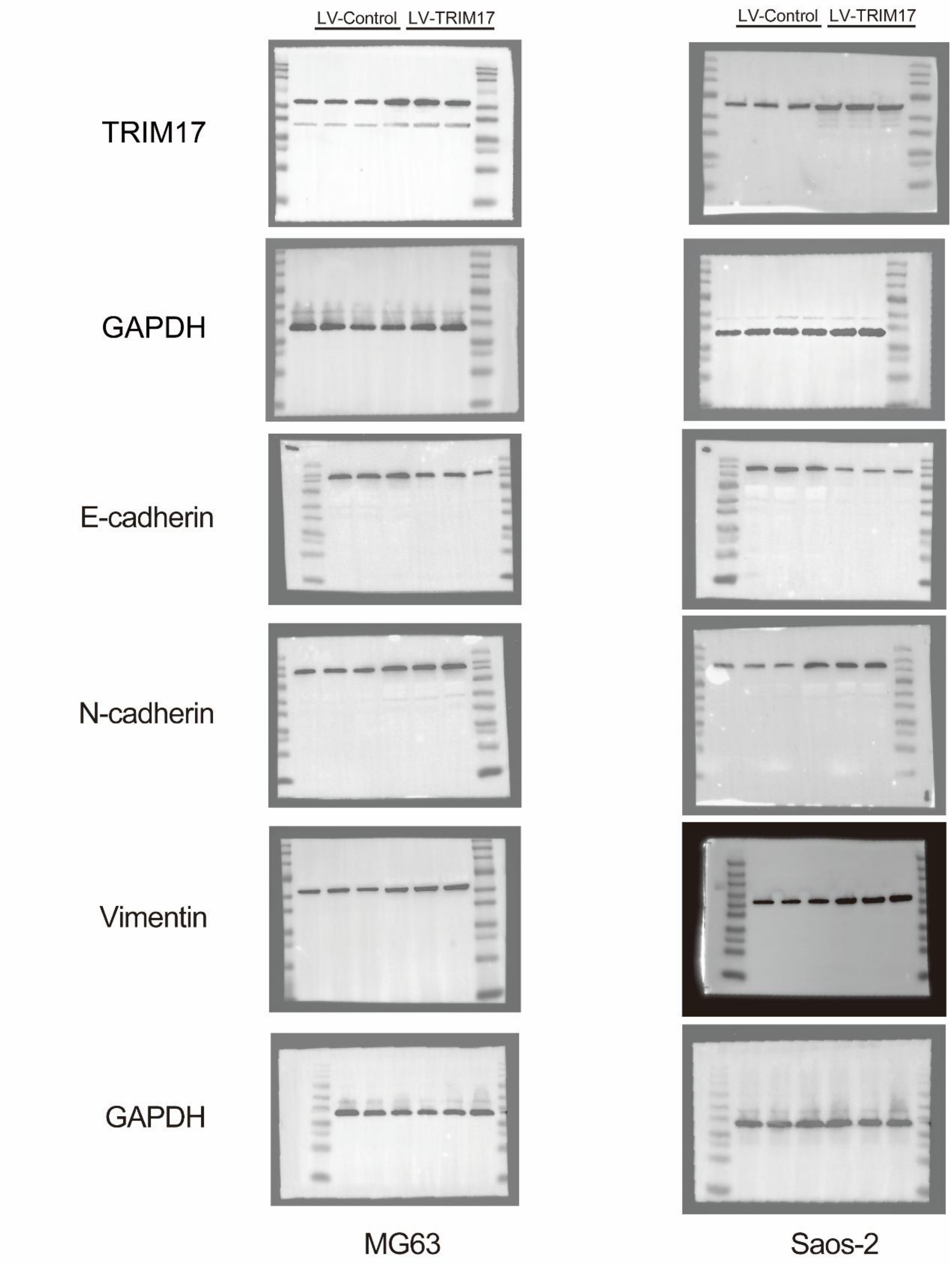

LV-Control
LV-TRIM17
LV-Control
LV-TRIM17
TRIM17
GAPDH
E-cadherin
N-cadherin
Vimentin
GAPDH
MG63
Saos-2

Figure4

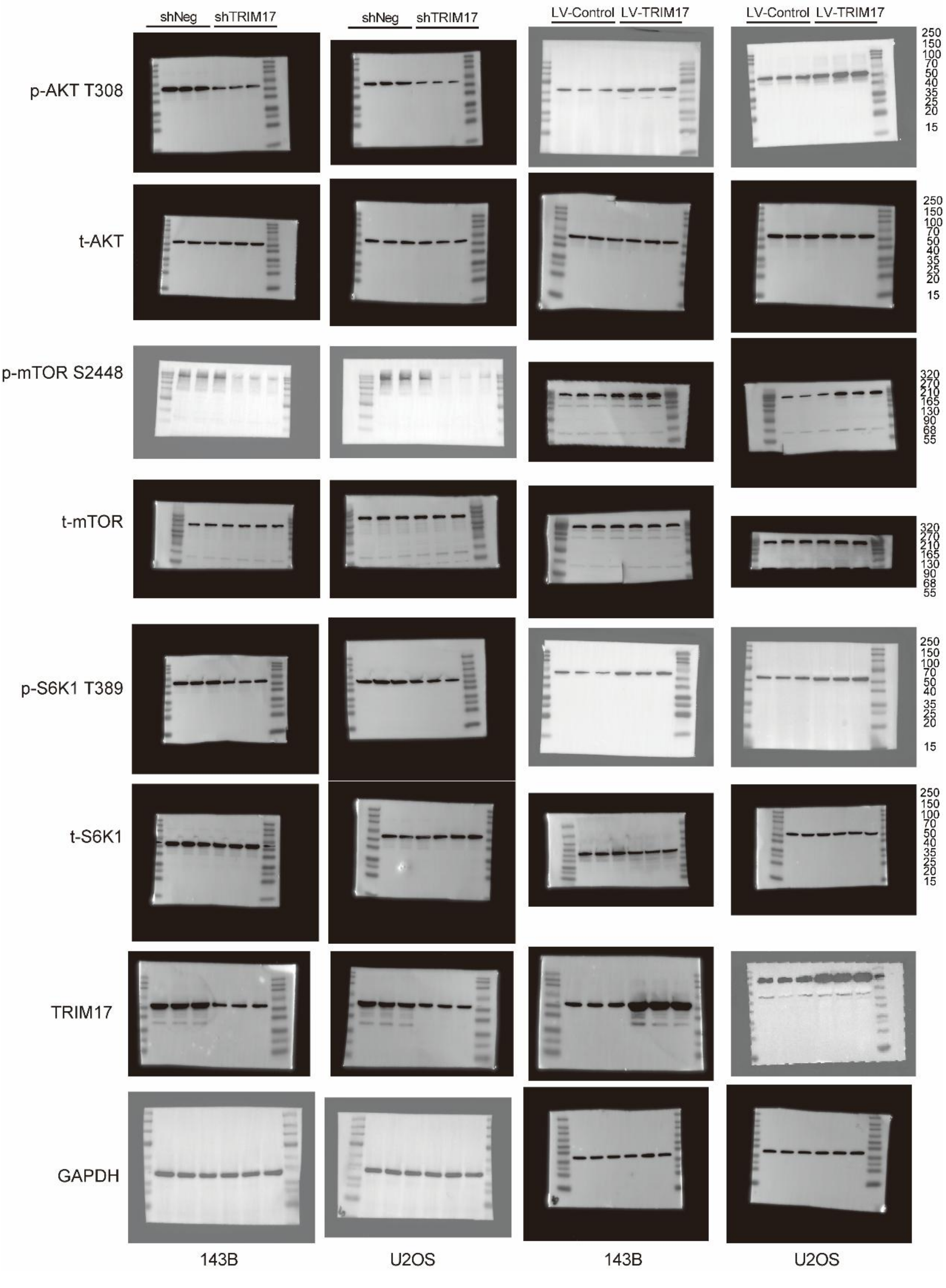
shNeg
shTRIM17
shNeg
shTRIM17
LV-Control
LV-TRIM17
LV-Control
LV-TRIM17
p-AKT T308
t-AKT
p-mTOR S2448
t-mTOR
p-S6K1 T389
t-S6K1
TRIM17
GAPDH
250
150
100
70
50
40
35
25
20
15
320
270
210
165
130
90
68
55
143B
U2OS
143B
U2OS

Figure5

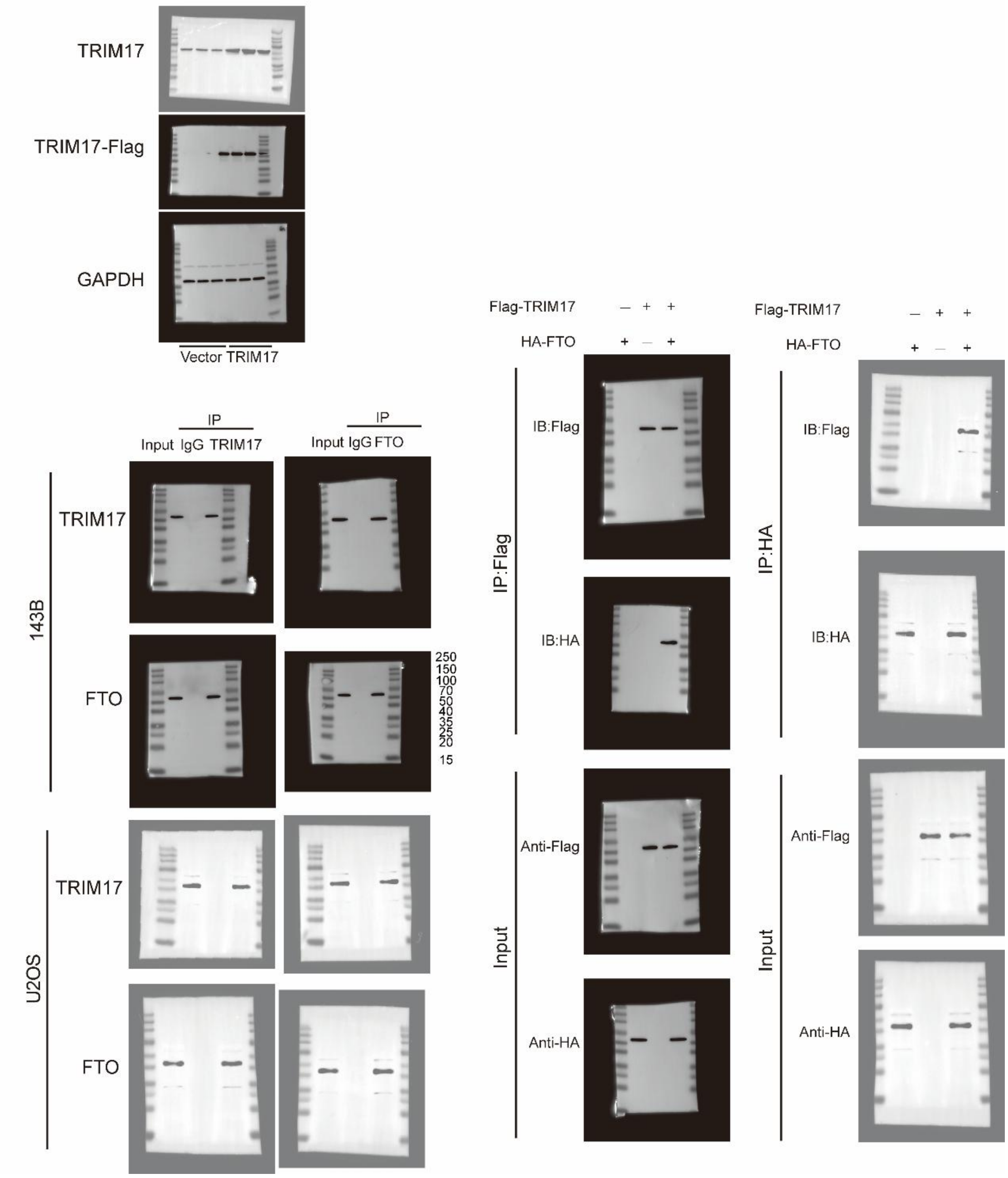
TRIM17
TRIM17-Flag
GAPDH
Vector
TRIM17
IP
Input
IgG
TRIM17
IP
Input
IgG
FTO
143B
TRIM17
FTO
250
150
100
70
50
40
35
25
20
15
U2OS
TRIM17
FTO
Flag-TRIM17
— + +
HA-FTO
+ — +
IP:Flag
IB:Flag
IB:HA
Input
Anti-Flag
Anti-HA
Flag-TRIM17
— + +
HA-FTO
+ — +
IP:HA
IB:Flag
IB:HA
Input
Anti-Flag
Anti-HA

Figure5

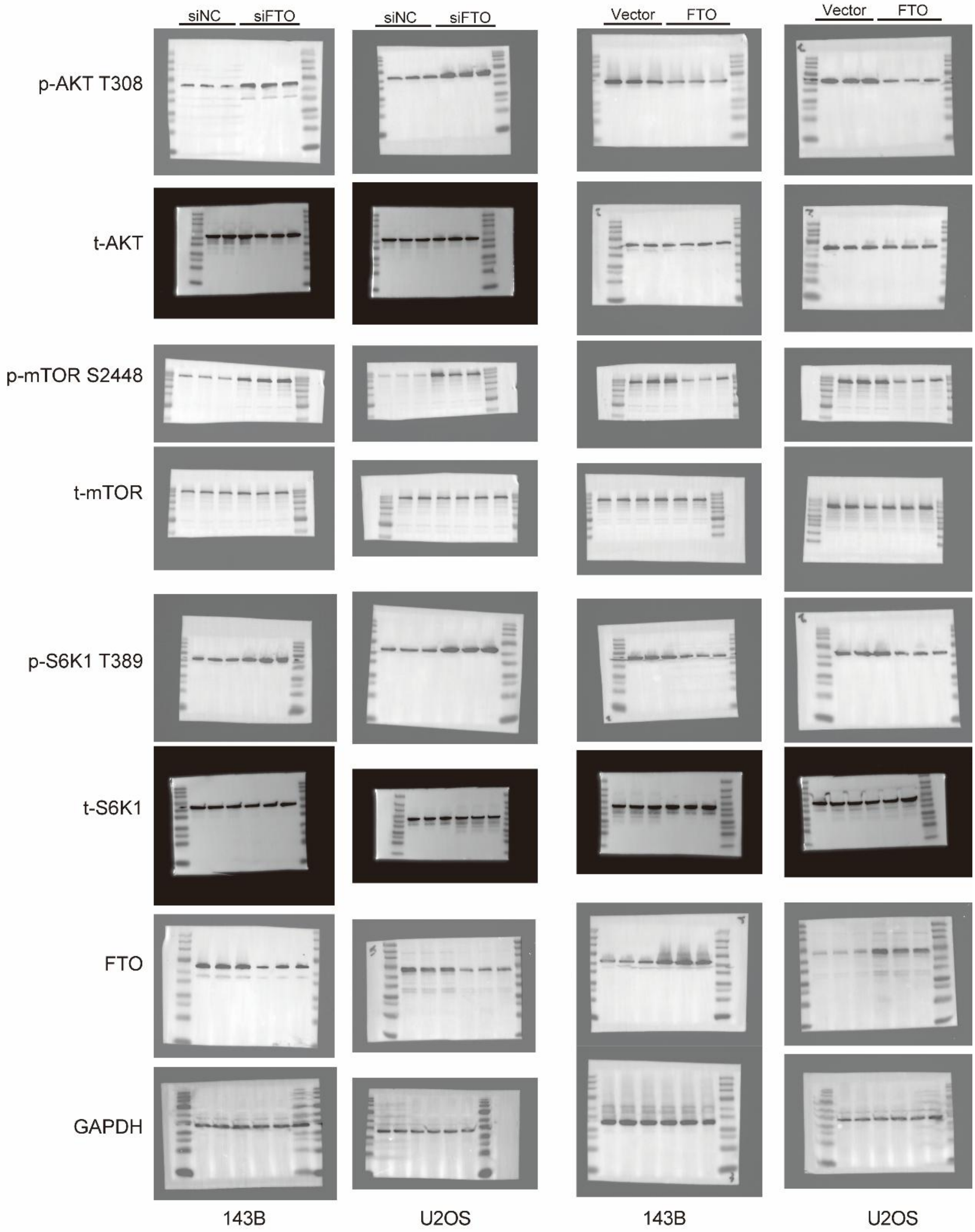

siNC
siFTO
siNC
siFTO
Vector
FTO
Vector
FTO
p-AKT T308
t-AKT
p-mTOR S2448
t-mTOR
p-S6K1 T389
t-S6K1
FTO
GAPDH
143B
U2OS
143B
U2OS

Figure6

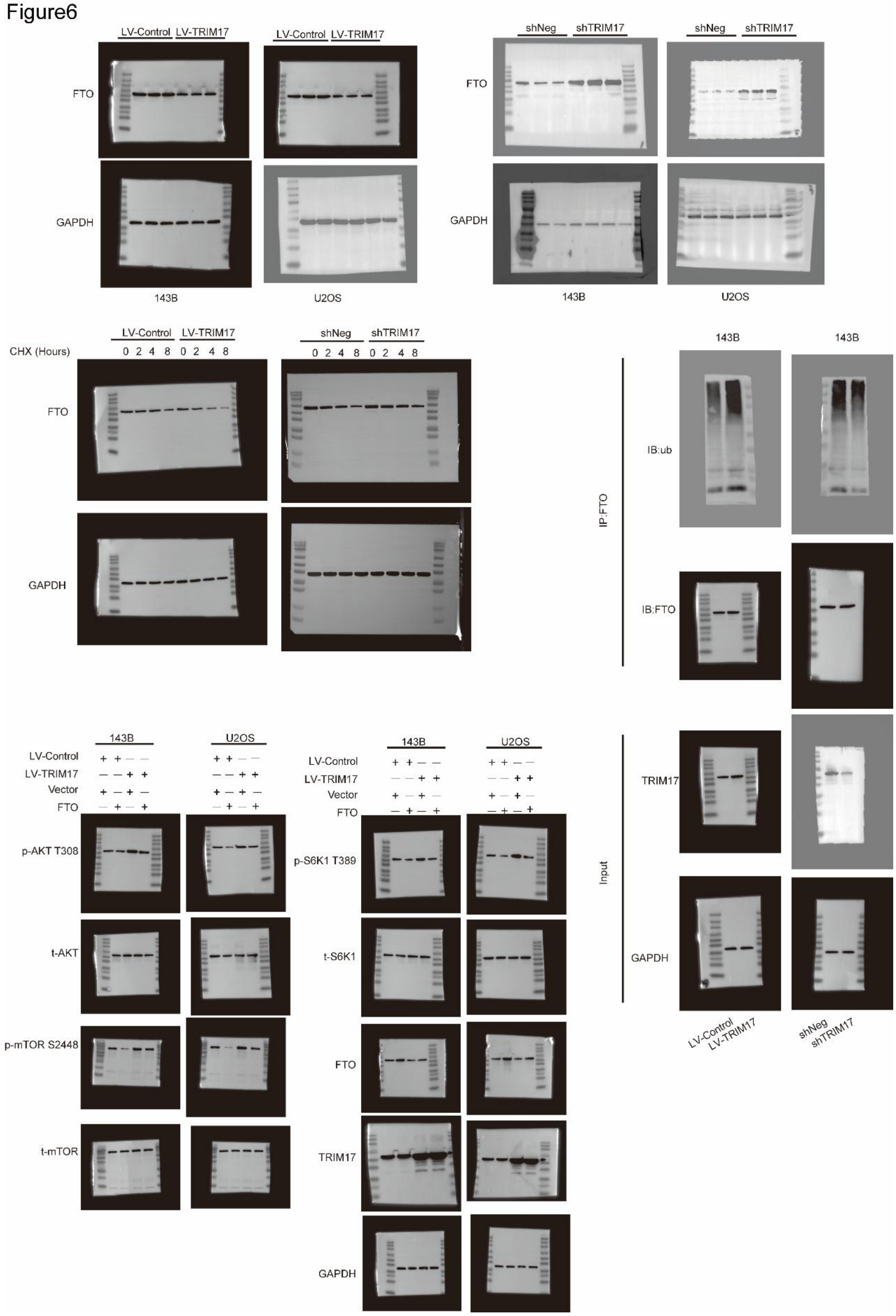

LV-Control
LV-TRIM17
shNeg
shTRIM17
FTO
GAPDH
143B
U2OS
CHX (Hours)
0 2 4 8 0 2 4 8
IP:FTO
IB:ub
IB:FTO
Input
TRIM17
LV-TRIM17
Vector
p-AKT T308
t-AKT
p-mTOR S2448
t-mTOR
p-S6K1 T389
t-S6K1

Figure7

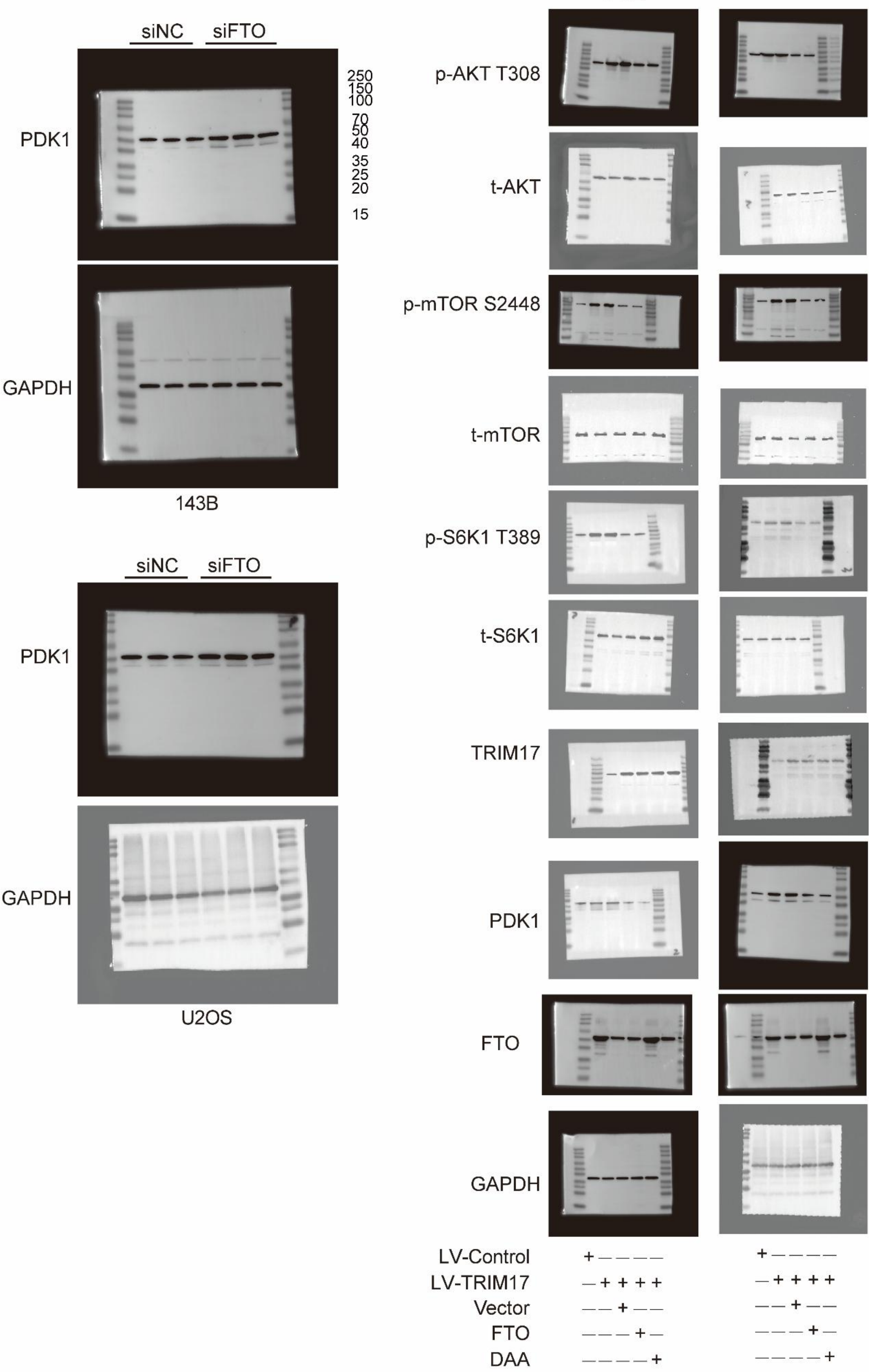
siNC
siFTO
PDK1
250
150
100
70
50
40
35
25
20
15
GAPDH
143B
siNC
siFTO
PDK1
GAPDH
U2OS
143B
U2OS
p-AKT T308
t-AKT
p-mTOR S2448
t-mTOR
p-S6K1 T389
t-S6K1
TRIM17
PDK1
FTO
GAPDH
LV-Control + – – – – + – – – –
LV-TRIM17 – + + + + – + + + +
Vector – – + – – – – + – –
FTO – – – + – – – – + –
DAA – – – – + – – – – +

Figure8

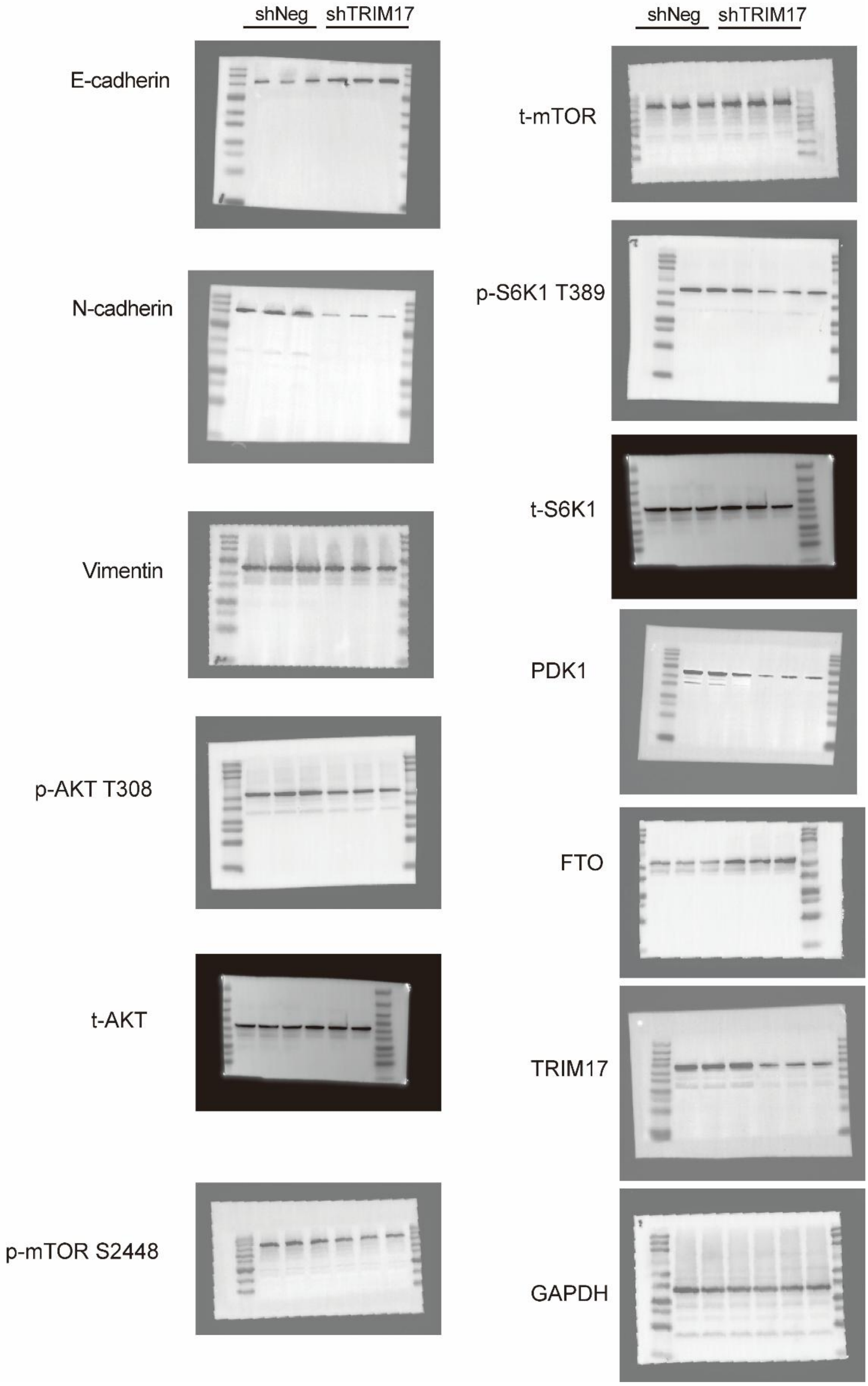

shNeg
shTRIM17
E-cadherin
N-cadherin
Vimentin
p-AKT T308
t-AKT
p-mTOR S2448
shNeg
shTRIM17
t-mTOR
p-S6K1 T389
t-S6K1
PDK1
FTO
TRIM17
GAPDH

Supplementary Figure S2

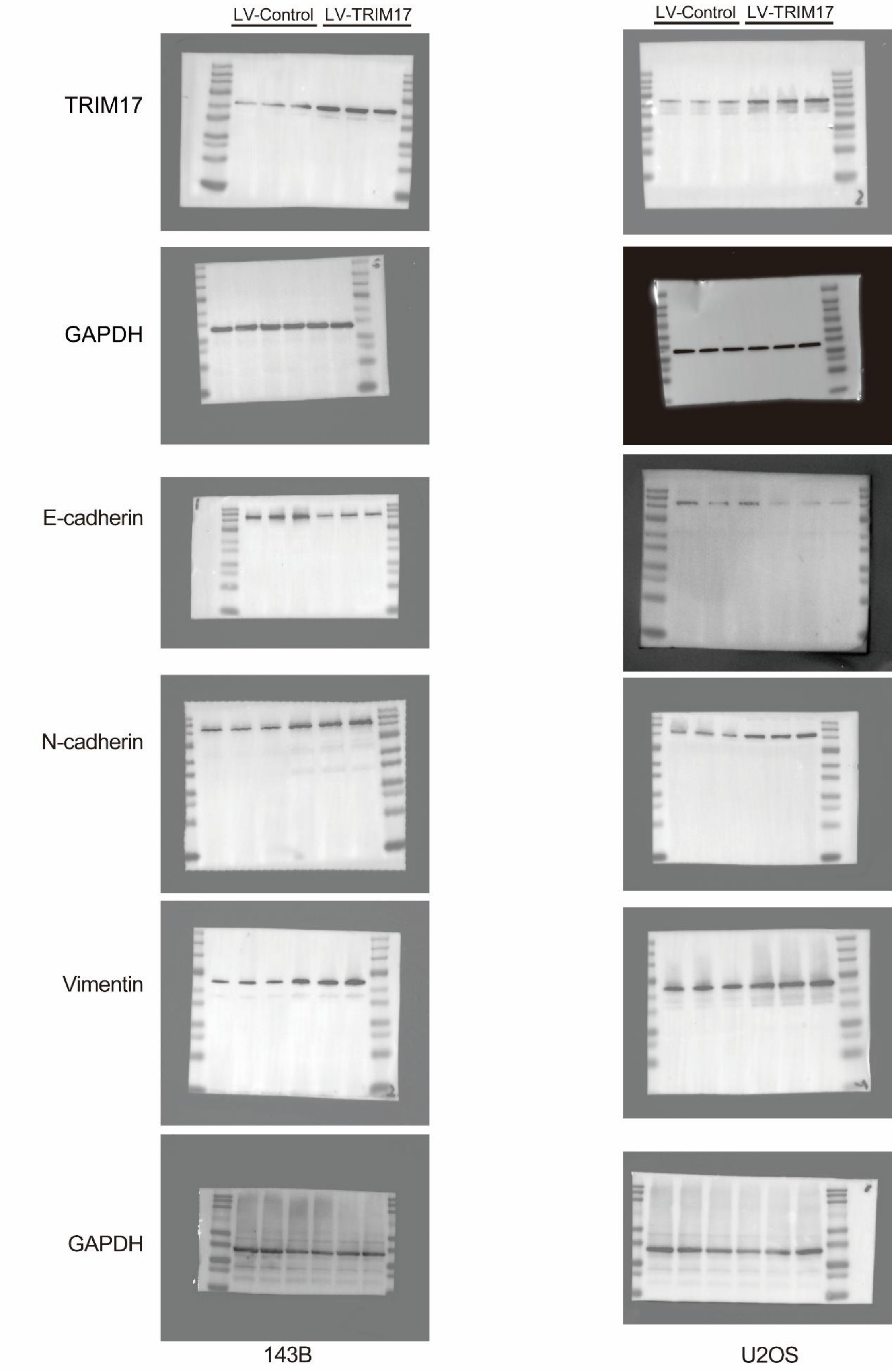

Supplementary Figure S3

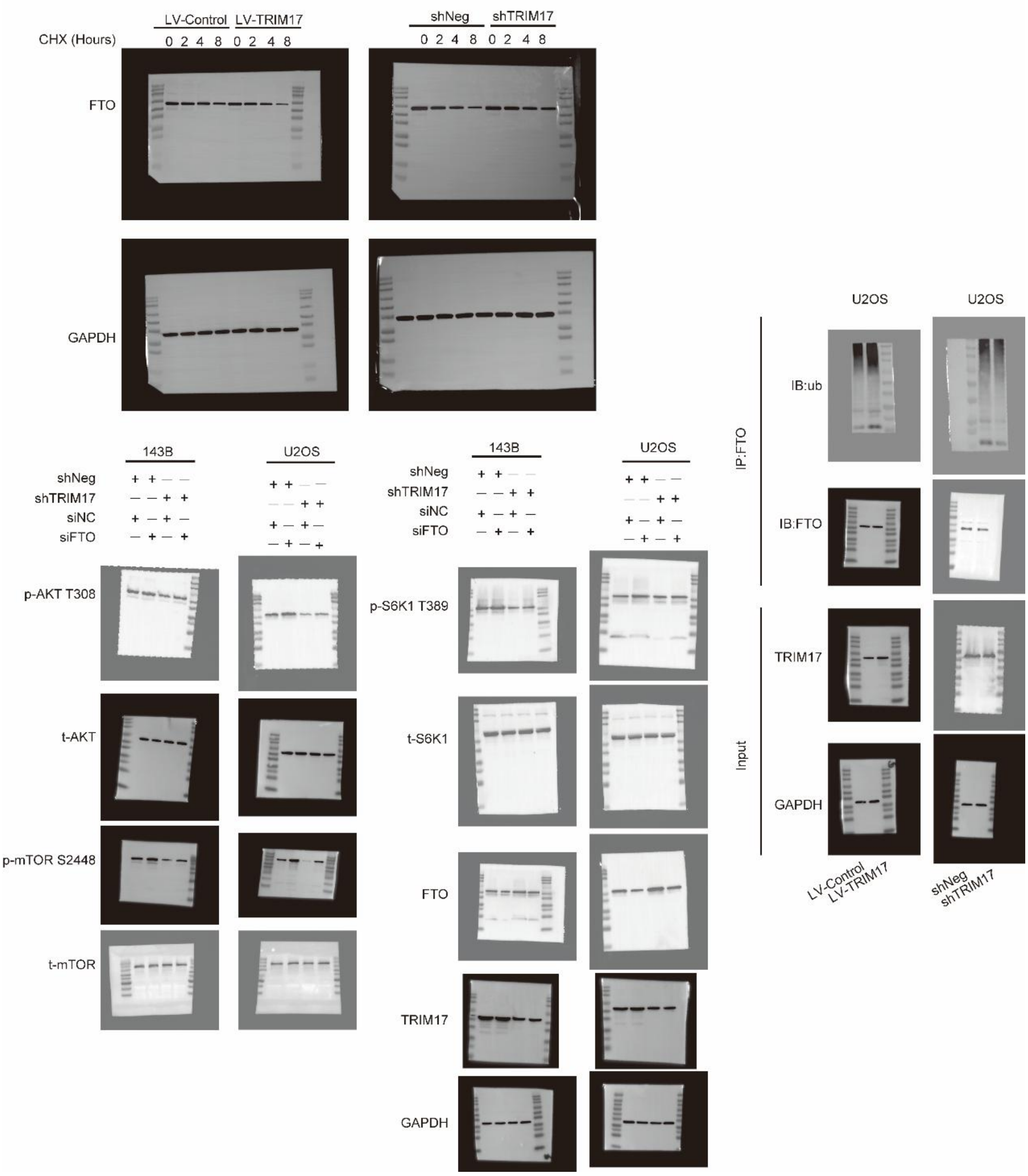

Supplement: Supplementary file 8 — Original blot [file 41419_2025_8070_MOESM8_ESM.pdf]
